# A Vital Signs Fast Detection and Extraction Method of UWB Impulse Radar Based on SVD

**DOI:** 10.3390/s22031177

**Published:** 2022-02-04

**Authors:** Siyun Liu, Qingjie Qi, Huifeng Cheng, Lifeng Sun, Youxin Zhao, Jiamei Chai

**Affiliations:** Emergency Research Institute, China Coal Research Institute CCRI, Beijing 100000, China; liusiyun1019@163.com (S.L.); feng20050508@163.com (H.C.); sunlifeng_2005@126.com (L.S.); 18531831279@163.com (Y.Z.); cjm7980@163.com (J.C.)

**Keywords:** ultra-wideband radar (UWB), vital sign extraction, singular value decomposition (SVD), wavelet transform decomposition, energy entropy, temporal and spatial eigenvectors

## Abstract

The identification of weak vital signs has always been one of the difficulties in the field of life detection. In this paper, a novel vital sign detection and extraction method with high efficiency, high precision, high sensitivity and high signal-to-noise ratio is proposed. Based on the NVA6100 pulse radar system, the radar matrix which contains several radar pulse detection signals is received. According to the characteristics of vital signs and radar matrices, the Singular Value Decomposition (SVD) is adopted to perform signal denoising and decomposition after preprocessing, and the temporal and spatial eigenvectors of each principal component are obtained. Through the energy proportion screening, the Wavelet Transform decomposition and linear trend suppression, relatively pure vital signs in each principal component, are obtained. The human location is detected by the Energy Entropy of spatial eigenvectors, and the respiratory signal and heartbeat signal are restored through a Butterworth Filter and an MTI harmonic canceller. Finally, through an analysis of the performance of the algorithm, it is proved to have the properties of efficiency and accuracy.

## 1. Introduction

In recent years, emergencies such as wars and terrorist attacks, natural disasters such as earthquakes and tsunamis and potential nuclear, chemical and biological dangerous goods and explosives have seriously endangered the safety of human life and property. Through years of experience in post-disaster rescue, domestic and foreign experts have concluded that 72 h after a disaster is the golden rescue time [1]. In this period, the primary task of rescue is to find and save the trapped victims as soon as possible, so as to minimize casualties. The severe situation puts forward very high requirements for life detection technology and equipment. It has become the focus of research in various countries to use advanced technology and equipment to rescue victims who survived the disaster better and faster without being seen by the naked eye.

Ultra-wideband radar is usually defined as a radar whose transmitted signal has a fractional bandwidth (FBW) greater than 0.25 [2]. It has the advantages of good electromagnetic compatibility, strong penetration of non-metallic media, high range resolution and low average transmitting power. The radar life detector is developed according to the reflection principle of electromagnetic waves. David Cist, a famous physicist and doctor of MIT, creatively applied radar ultra-wideband technology (UWB) to the field of safety rescue, thus bringing a revolutionary new technology to this field [3,4]. It detects various micro-movements caused by human life activities and obtains relevant information about respiration and heartbeats from those micro-movements, thereby identifying the survivors [5]. However, there are many factors that will cause strong interference and even cover up the useful weak signals, such as the direct wave and strong clutter formed by multiple reflections off walls, the coupled signal of objects and antennas, the thermal noise caused by the passive components in the circuit, the linear trend caused by DC components, etc. [6,7,8]. Therefore, weak signal detection has always been a hotspot and a vexed issue in the field of signal processing.

Among traditional denoising methods, the Median Filter and the Wiener Filter cannot describe the non-stationary characteristics of the signal [9]. The Time-domain Mean Subtraction method can remove background clutters of stationary targets, but it will inevitably bring additional harmonic interference [10,11]. The Linear Trend Subtraction method can effectively remove static clutters and linear trend items of echo [12]. The Auto Gain Control method can enhance weak vital signs, thereby further improving the signal-to-noise ratio [13]. Gerstein used the high-order cumulant method to detect the heartbeat signal, which can effectively suppress Gaussian noise [14]. However, the above-mentioned traditional methods can only suppress specific components in the UWB echo signal, which is difficult to meet the demand for the accurate detection of weak vital signs.

Since Fourier Transform was proposed in 1807, it has played an irreplaceable role in the field of signal processing [15]. The Wavelet Transform theory, as a time-domain analysis method developed by inheriting the Fourier Transform, has gradually emerged and has been applied to the extraction technology of vital signs in recent years [16]. Wavelet Transform can perform a time-frequency joint analysis of signals, with high resolution, and can effectively denoise and extract non-stationary signals [17,18]. Kumar used Wavelet Transform to process ECG signals with an improved threshold algorithm to remove the influence of baseline drift, power frequency interference and EMG interference [19]. Guevorkian applied the pipeline structure to the discrete Wavelet Transform, which greatly improved the computational efficiency [20]. Liu Xiuping used Wavelet Transform and Lifting Wavelet Transform to denoise the X-ray pulsar signals polluted by large noises [21]. Yang Xiufang used a soft-threshold function and hard-threshold function to denoise the radar signal under strong noise interference [22].

In recent years, the Empirical Mode Decomposition (EMD) method has attracted the attention of scholars because it completely got rid of the limitations of time-frequency analysis [23,24]. Based on the EMD method, Xiong filtered each of the decomposed IMF components and reconstructed the denoised signal [25]. In 2017, Giovanni proposed a method of extracting heartbeat and respiratory signals by using the EMD method in a certain range combined with Doppler information [26]. Yan J. of Nanjing University of Science and Technology used the Variational Mode Decomposition (VMD) method to extract the vital signs of one or more targets based on UWB radar [27]. In 2018, in the vital signs monitoring system based on UWB radar proposed by Liang, the spectrum accumulation of Ensemble Empirical Mode Decomposition (EEMD) was used to detect the vital signs parameters, which improved the performance and detection accuracy of the EMD method [28].

In addition, Spatial Spectrum Estimation is also an emerging technology in the field of signal processing in recent years [29,30,31]. Since the signal space of heartbeats and respiration is orthogonal to the noise signal space, a Multiple Signal Classification (MUSIC) algorithm can be used for spectrum estimation [32,33,34]. Z. Dong applied the Root-MUSIC algorithm to greatly improve the performance of the MUSIC algorithm, reduce the amount of calculation and improve the efficiency of vital sign extraction [35]. Other effective methods include ARMA Model Estimation [36,37], Maximum Likelihood Estimation [38], Entropy Spectrum Estimation [39,40], Pisarenko Harmonic Decomposition [41], Eigen Decomposition Method [42,43,44], etc.

At present, during the actual disaster rescue process, UWB life detectors have played a certain role, but it is undeniable that the application effect is still limited. In this paper, a new method is proposed to improve the robustness, accuracy and timeliness of the UWB radar signal processing and vital signs extraction algorithm. Thus, the life detector can better detect the weak life signals in various complex environments and find the trapped survivors more accurately and quickly.

## 2. Basic Principles of UWB Life Detection

The UWB radar life detector detects the situation inside the ruins by transmitting pulse detection signals to the surface. Based on the time-domain Doppler effect of vital signs within the UWB radar echo signals, it analyzes whether there are survivors in the ruins and calculates the specific location information. When a UWB radar signal encounters the trapped victims and generates an echo signal, the human life activities change the parameters of the echo signal. This modulation effect on the echo signal has the following characteristics:

(1) The vital signs of the human body such as respiration and heartbeat have low-frequency characteristics. The human heartbeat is about 70–80 times/min, and respiration is about 20–40 times/min. Therefore, the modulation frequency of vital signs to radar echo signals is in the range of 0.2 to 2 Hz.
(2) Under normal circumstances, human vital signs have relatively accurate periodic characteristics.

**Figure 1:**
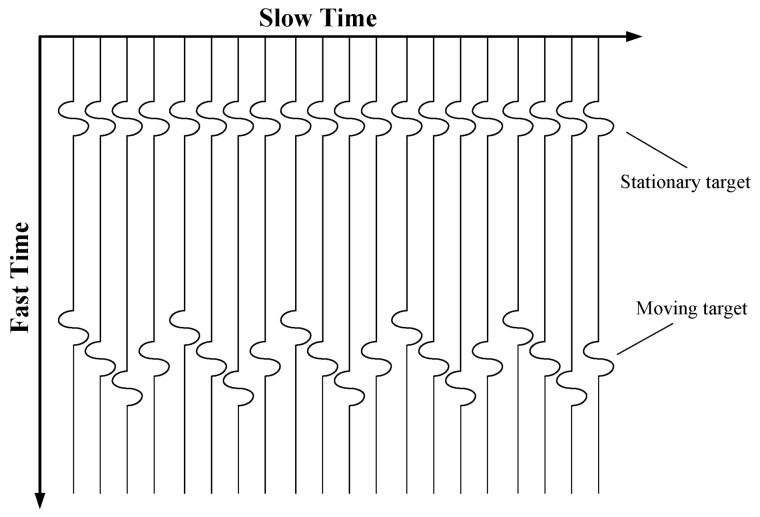
Schematic diagram of vital signs signals detection effect.

Figure 1 shows the characteristics of the echo signals of stationary targets and human periodic fretting vital signs. Assuming that the fluctuating motion of the chest caused by respiration and heartbeat changes in the law of sinusoidal signals, the distance between the vital signs’ signal source and the UWB radar detector can be expressed as follows:

(1) $$d(t)=d_{0}+\Delta d_{r}\sin2\pi f_{r}t+\Delta d_{h}\sin2\pi f_{h}t,$$

where *d*(*t*) represents the distance from chest to radar antenna, *d*_0_ is the average reference distance from chest to radar antenna, Δ*d_r_* is the amplitude of chest changes caused by respiration, *f_r_* is the respiration frequency, Δ*d_h_* is the amplitude of chest changes caused by the heartbeat and *f_h_* is the heartbeat frequency. In the process of vital signs detection, the response caused by the radar pulse signal is the superposition of echo signals of static objects and dynamic objects:

(2) $$h(t,\tau )=\sum_{i}\alpha_{i}\delta (\tau -\tau_{i})+\alpha_{d}\delta (\tau -\tau_{d}(t)),$$

(3) $$\tau_{d}(t)=\frac{2d(t)}{c}=\tau_{0}+\tau_{r}\sin2\pi f_{r}t+\tau_{h}\sin2\pi f_{h}t,$$

where the former represents the static response generated in the detection environment, and the latter represents the dynamic response caused by human respiration and heartbeat. Moreover, *α_d_* is the amplitude of the dynamic response, and *τ_d_*(*t*) is the time delay of dynamic response. In addition, it can be seen from Equation (3) that the motion of the chest cavity modulates the echo signal, resulting in a certain periodic change in the position of the dynamic time delay. Therefore, the echo signal received by the receiving antenna can be regarded as the convolution of the transmitted pulse signal and the response function, which is expressed by the following equation:

(4) $$\begin{array}{l} r(t,\tau )=p(t)*h(t,\tau )\\ =\sum_{i}\alpha_{i}p(\tau -\tau_{i})+\alpha_{d}p(\tau -\tau_{d}(t)), \end{array}$$

where *p*(*t*) is the radar pulse detection signal. In Equation (4), *t* represents the accumulation time of multiple pulses transmitted by the radar, and *τ* represents the sampling time of each pulse. The pulse sampling time is closely related to the distance information from each position in space to the radar antenna.

Sampling the radar signals’ waveform at continuous discrete time series when the pulse accumulation time *t* = *mT_s_* (*m* = 1, 2, …, *M*), the received echo signals observed for a long time are:

(5) $$r(mT_{s},\tau )=\sum_{i}\alpha_{i}p(\tau -\tau_{i})+\alpha_{d}p(\tau -\tau_{d}(mT_{s})),$$

Storing the discrete time series of *M* pulse signals after sampling, namely, the UWB pulse echo signals are discretized and stored in a two-dimensional matrix of *N* rows and *M* columns, the UWB echo matrix is formed:

(6) $$\begin{array}{ll} R_{N\times M} & =\left\{r[n,m]\right\}\\ & =\left\{\sum_{i}\alpha_{i}p(n\delta_{\tau }-\tau_{i})+\alpha_{d}p(n\delta_{\tau }-\tau_{d}(mT_{s}))\right\}\;,\\ & =\left\{c[n]+h[n,m]\right\}\;(1\leq m\leq M,\;1\leq n\leq N) \end{array}$$

where *T_s_* is the pulse repetition interval time, *δ_τ_* is the sampling interval in fast time, *m* is the number of slow time samples, *n* is the number of fast time samples, *c*[*n*] is the echo signals components of static object and *h*[*n*, *m*] is the vital signs of respiration and heartbeat.

Based on the above analysis, one can preliminarily judge whether there are vital signs by filtering the static object components and detecting whether there are periodic change characteristics in the UWB echo signals.

## 3. Construction of Vital Signs Detection System

**Figure 2:**
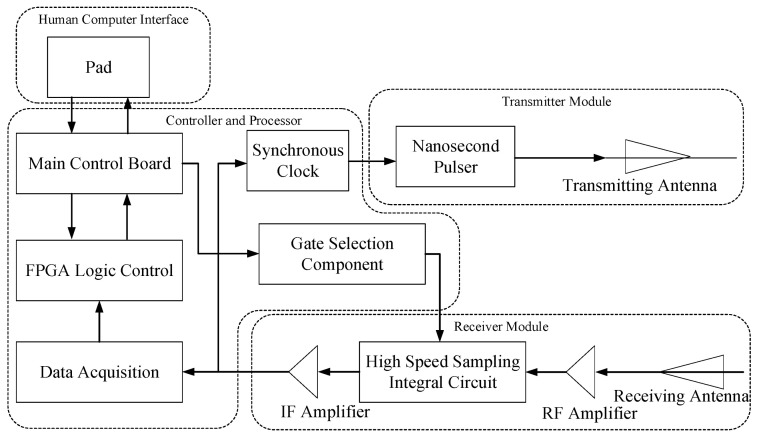
Hardware structure of the vital signs detection system.

**Figure 3:**
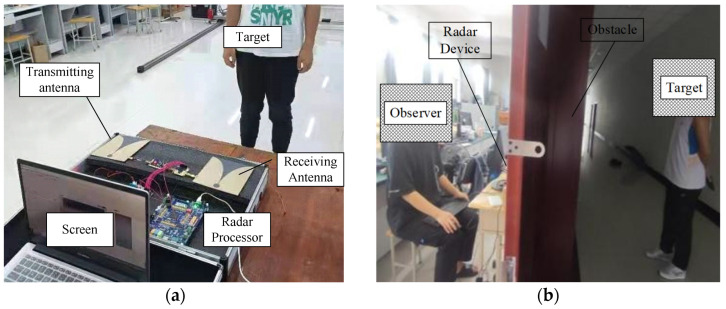

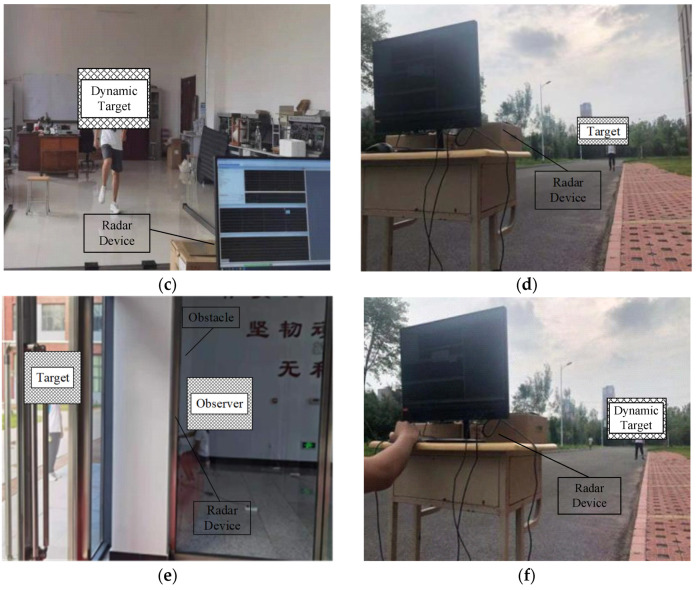
Experimental setup of the system for different scenes: (**a**) Detection of indoor stationary target; (**b**) Detection of indoor stationary target covered by obstacle; (**c**) Detection of indoor dynamic target; (**d**) Detection of outdoor stationary target; (**e**) Detection of outdoor stationary target covered by obstacle; (**f**) Detection of outdoor dynamic target.

In this project, the NVA6100 pulse radar experimental platform is adopted. Two transceiver antennas based on the Vivaldi antenna structure are used to realize the transmission and reception of UWB signals, and the transmitted signals are approximately first-order Gaussian pulse signals. There are two independent pulse generators inside the NVA6100 chip, which can generate nanosecond pulse detection signals with high range resolution. Figure 2 shows the hardware structure of the vital sign detection system. Through setting the internal registers, the detection, acquisition and processing of UWB echo signals can be realized. The fast time domain sampling frequency is set to 39 GHz, the slow time domain sampling frequency is set to 152.6 Hz and the fast time domain sampling length is 512. Figure 3 shows the experimental setup of the system for different scenes. According to the actual test environment, a simple test system platform is built on site to detect the performance of the radar device in practical application. An NVA6100 Radar device is used for UWB radar signal transmission, data acquisition and processing. A computer is used to receive and store data and display the algorithm running results. While the radar device is detecting, the detected target person records the number of breaths he takes during the test period, and the heart rate tester is used to test the heartbeat frequency. In order to test whether the algorithm proposed in this paper can meet the actual needs, different test scenes, such as the detection of indoor stationary targets, targets covered by obstacles, dynamic targets, multiple targets and targets in complex outdoor environments are constructed. Figure 3a shows the indoor obstacle free detection scene; Figure 3b shows the indoor obstacle crossing the detection scene, where the obstacle is a wall made of concrete with a thickness of 25 cm and a density of 2500 kg/m^3^; Figure 3c shows the indoor dynamic object detection sense where the dynamic target is marching on the spot; Figure 3d shows the outdoor obstacle-free detection scene; Figure 3e shows the outdoor obstacle crossing detection scene, the radar device need to pass through the obstacle to detect the outdoor human target, and the obstacle is a wall made of concrete with a thickness of 32 cm and a density of 2500 kg/m^3^; Figure 3f shows the outdoor dynamic object detection sense where the dynamic target is marching on the spot.

**Figure 4:**
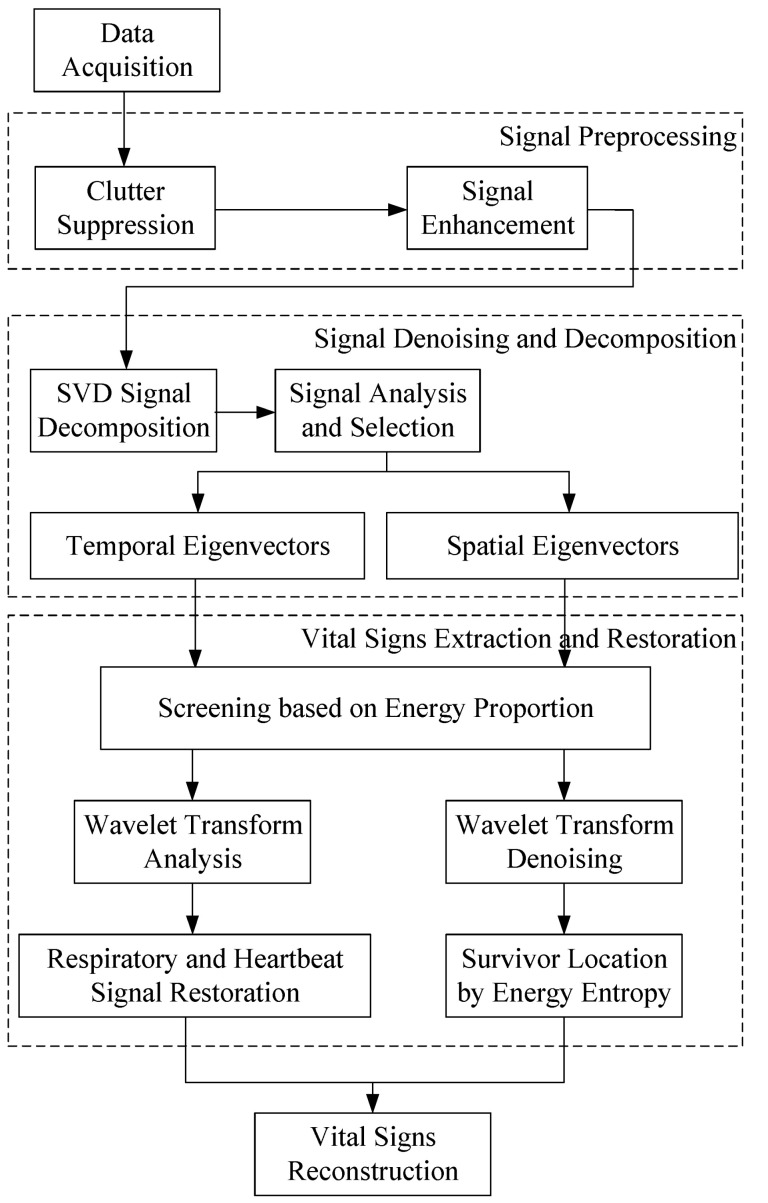
Specific flowchart of proposed algorithm.

In a complex field detection environment, weak respiration and heartbeat signals are covered by a large number of noises and clutters. Therefore, in order to improve the accuracy of vital signs extraction in low signal-to-noise ratio environment, the algorithm proposed in this paper includes the following steps: signal preprocessing, signal denoising and decomposition, vital signs extraction and restoration, and vital signs reconstruction. Figure 4 shows the specific flowchart of the algorithm proposed in this paper. In the following chapters, firstly, the algorithm will be explained in detail with the sense of indoor stationary object detection, which is regarded as the simplest case. Then, the experimental results in other groups of scenarios will be displayed to verify the effectiveness of the proposed method in the actual complex environment. Finally, the performance of the proposed algorithm will be compared with the existing technologies to illustrate the advancement of the method.

## 4. Signal Preprocessing

Under the actual detection circumstances, there are a lot of interference clutters in the UWB echo signal. The echo matrix model in a real environment is shown as follows:

(7) R[n,m]=h[n,m]+c[n]+w[n,m]+d[m]+l[n,m],

where *h*[*n*, *m*] represents the vital signs to be acquired, *c*[*n*] represents the static background clutters, *w*[*n*, *m*] represents the additive white noises caused by the radar system source, *d*[*m*] represents the unstable fast time DC components and *l*[*n*, *m*] is the linear trend on the slow time axis due to the unstable amplitude of the radar system during the acquisition process. After obtaining the UWB echo signals, it is necessary to remove some above clutter through signal preprocessing.

### 4.1. Clutter Suppression

Background clutters *c*[*n*] refers to all static components of signals unrelated to vital signs. They are generated from the multi-path reflection of human limbs, trunks and static objects, thus hardly any change can be observed in fast time.

#### 4.1.1. Range Profile Subtraction (RPS) Method

The Background clutters *c*[*n*] always contain high-frequency components. Static background clutter which has a large amplitude can be regarded as a constant component for the scanning process. To perform the pulse cancellation, the Range Profile Subtraction (RPS) method is applied:

(8) $$\begin{array}{l} R^{\prime }[1,m]=R[1,m]\\ R^{\prime }[n,m]=R[n,m]-R[n-1,m]\begin{array}{cc} & \left(m=1,\ldots ,M,\;n=2,\ldots ,N\right) \end{array} \end{array},$$

#### 4.1.2. Time Mean Subtraction (TMS) Method

The background clutter caused by detecting static objects in the scene can be approximated as a DC component, so the Time Mean Subtraction (TMS) method is used to eliminate this component:

(9) $$R^{\prime \prime }[n,m]=R^{\prime }[n,m]-\frac{1}{N}\sum_{i=1}^{N}R^{\prime }[i,m]\begin{array}{cc} & \left(m=1,\ldots ,M,\;n=1,\ldots ,N\right) \end{array},$$

### 4.2. Signal Enhancement

In the actual application scene of life detectors, UWB pulse signals often need to penetrate walls, ruins, sheltered objects and other media, which often leads to the concealment of key vital signs. At the same time, the strength of the received signal is related to the signal transmission distance, so the echo signal of the distant target object is relatively weak. In view of the above situation, the weak signal enhancement algorithm for the original radar echo signals is the key to improve the detection success rate in a complex environment.

#### 4.2.1. Advance Normalization Method

To enhance the vital signs, the Advance Normalization method is performed. The overall idea of the algorithm is to perform piecewise normalization for the whole signal interval according to the multi-order maximum, so as to strengthen the key information masked by the previous large amplitude signals. The specific flow of the Advance Normalization method is as follows:

In the first iteration step, for each column of echo signal {*R*″[*i*, *m*], *i* = 1, …, *N*}, find the maximum value *S*_max_(1) of the echo sampling data. *τ*_max_(1) is the position of the sampling point corresponding to the maximum value *S*_max_(1). According to Equation (10), for *i* = 1, …, *τ*_max_(1), the echo data are normalized with *S*_max_(1):

(10) $$\begin{array}{l} S_{\max}(1)=R^{\prime \prime }[\tau_{\max}(1),m]=\mathrm{Max}(R^{\prime \prime }[i,m])\begin{array}{cc} & i=1,\ldots ,N \end{array}\\ Y[i,m]=\frac{R^{\prime \prime }[i,m]}{S_{\max}(1)}=\frac{R^{\prime \prime }[i,m]}{R^{\prime \prime }[\tau_{\max}(1),m]}\begin{array}{cc} & i=1,\ldots ,\tau_{\max}(1) \end{array} \end{array},$$

In subsequent iteration steps, assuming that the iteration step number is *j*, partial echo sampling data {*R*″[*i*, *m*], *i* = *τ*_max_(*j* − 1) + 1, …, *N*} are intercepted. Find the *j* order maximum value *S*_max_(*j*). *τ*_max_(*j*) is the position of the sampling point corresponding to the maximum value *S*_max_(*j*). According to Equation (11), for *i* = *τ*_max_(*j* − 1) + 1, …, *τ*_max_(*j*), the echo data are normalized with *S*_max_(*j*):

(11) $$\begin{array}{l} S_{\max}(j)=R^{\prime \prime }[\tau_{\max}(j),m]=\mathrm{Max}(R^{\prime \prime }[i,m])\begin{array}{cc} & i=\tau_{\max}(j-1)+1,\ldots ,N \end{array}\\ Y[i,m]=\frac{R^{\prime \prime }[i,m]}{S_{\max}(j)}=\frac{R^{\prime \prime }[i,m]}{R^{\prime \prime }[\tau_{\max}(j),m]}\begin{array}{cc} & i=\tau_{\max}(j-1)+1,\ldots ,\tau_{\max}(j) \end{array} \end{array},$$

The above iterative process continues until *τ*_max_(*j*) = *N*. The Advance Normalization process of the whole UWB echo signal is completed.

#### 4.2.2. Automatic Gain Control (AGC) Method

Automatic Gain Control is an automatic control method that makes the gain of the amplification circuit automatically adjust with the signal strength. It takes the effective combination of linear amplification and compression amplification to adjust the output signal. Therefore, the Automatic Gain Control (AGC) method is used to enhance the weak vital signs in slow time, so as to further improve the signal-to-noise ratio. The specific algorithm is as follows:

First, set the sliding window length *d* and the maximum gain value *g*_max_. Then, calculate the corresponding gain coefficient according to the signal power within the window. Finally, calculate the controlled data by multiplying the gain coefficient to realize the adaptive control effect:

(12) $$g(i,m)=\frac{2d+1}{\sqrt{\sum_{k=i-d}^{i+d}Y{\left(i,m\right)}^{2}}},$$

(13) $$g_{mask}(i,m)=\{\begin{array}{c} g_{\max}\\ g(i,m) \end{array}\begin{array}{c} ,g(i,m)>g_{\max}\\ ,g(i,m)\leq g_{\max} \end{array},$$

(14) $$X(i,m)=g_{mask}(i,m)Y(i,m),$$

**Figure 5:**
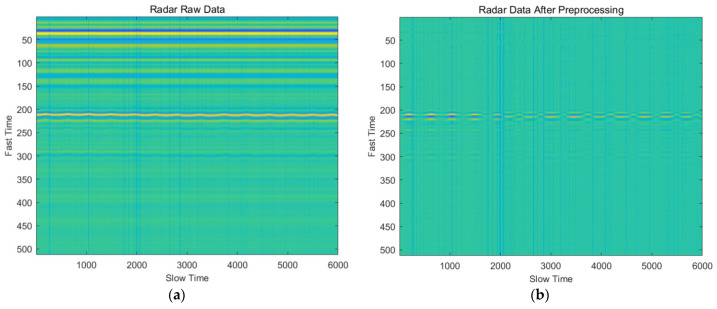
Signal preprocessing performance: (**a**) Radar raw data; (**b**) Radar data after preprocessing.

Figure 5 shows the radar data before and after preprocessing.

## 5. Signal Processing Based on SVD

### 5.1. Singular Value Decomposition Principle

Singular Value Decomposition (SVD) is a widely used algorithm in the field of machine learning. It can be used for characteristic decomposition and massive data compression. In this paper, the SVD method is used to process the radar data after signal preprocessing, so as to achieve the purpose of denoising, data compression, dimensionality reduction and characteristic decomposition. The steps of Singular Value Decomposition (SVD) for the radar data *X_N_*_×*M*_, which is after signal preprocessing, will be described in detail later.

The process of SVD is to decompose the radar data matrix *X_N_*_×*M*_ into the following form:

(15) $$X_{N\times M}=U_{N\times N}\Sigma_{N\times M}V_{M\times M}^{T}=\sum_{i=1}^{k}u_{i}\sigma_{i}v_{i}{}^{T},$$

(16) $$\begin{array}{l} \Sigma_{N\times M}={\left[\begin{array}{cccccc} \sigma_{1} & 0 & 0 & 0 & 0 & 0\\ 0 & \sigma_{2} & 0 & 0 & 0 & 0\\ 0 & 0 & \sigma_{3} & 0 & 0 & 0\\ \vdots & \vdots & \vdots & \ddots & 0 & 0\\ 0 & 0 & 0 & 0 & \ddots & 0 \end{array}\right]}_{N\times M,}\\ \sigma_{1}\geq \sigma_{2}\geq \sigma_{3}\geq \cdots \geq \sigma_{k}\begin{array}{cc} & ,k\leq \min(N,M) \end{array} \end{array}$$

(17) $$U_{N\times N}=\left[{\vec{u}}_{1},{\vec{u}}_{2},{\vec{u}}_{3},\cdots {\vec{u}}_{N}\right],$$

(18) $$V_{M\times M}=\left[{\vec{v}}_{1},{\vec{v}}_{2},{\vec{v}}_{3},\cdots {\vec{v}}_{M}\right],$$

Firstly, the square matrices *XX^T^* and *X^T^X* are constructed. Since both *XX^T^* and *X^T^X* are real symmetric square matrices, they can be performed orthogonal similarity diagonalization. According to the principle of eigenvalue decomposition and the form after SVD, one can obtain:

(19) $$\begin{array}{ll} XX^{T} & =U\Sigma V^{T}{\left(U\Sigma V^{T}\right)}^{T}\\ & =U\Sigma V^{T}V\Sigma^{T}U^{T}=U(\Sigma \Sigma^{T})U^{T},\\ & =U\Sigma_{1}U^{T} \end{array}$$

(20) $$\begin{array}{ll} X^{T}X & ={\left(U\Sigma V^{T}\right)}^{T}U\Sigma V^{T}\\ & =V\Sigma^{T}U^{T}U\Sigma V^{T}=V(\Sigma^{T}\Sigma )V^{T},\\ & =V\Sigma_{2}V^{T} \end{array}$$

*U_N__×N_* and *V_M_*_×*M*_ are unit orthogonal matrices, namely, *UU^T^* = *I* and *VV^T^* = *I*. Σ_1_ and Σ_2_ are the eigenvalue matrices, which have singular values only on the main diagonal, and only zero value for any other element.

According to Equations (19) and (20), the eigenvector matrix *U_N__×N_* of *XX^T^* is the left singular matrix of *X_N_*_×*M*_ when performing SVD, and the eigenvector matrix *V_M_*_×*M*_ of *X^T^X* is the right singular matrix of *X_N_*_×*M*_ when performing SVD. The singular value matrix Σ_*N*×*M*_ can be obtained by extracting the square root of the eigenvalues of matrix Σ_1_ or matrix Σ_2_.

The singular value matrix Σ_*N*×*M*_ has singular values only on the main diagonal, namely, *σ*_1_, *σ*_2_, *σ*_3_, …, *σ_k_*, *σ*_1_ ≥ *σ*_2_ ≥ *σ*_3_ ≥ … ≥ *σ_k_*. *u_i_* represents the *i*th column vector of matrix *U_N__×N_*, which is called the *i*th order left singular vector. *v_i_* represents the *i*th column vector of matrix *V_M_*_×*M*_, which is called the *i*th order right singular vector. *σ_i_* represents the *i*th element of the singular value spectrum.

### 5.2. Data Denoising and Dimensionality Reduction Based on SVD

According to the principle of principal components analysis, for radar data *X_N_*_×*M*_, each column vector *x_i_* represents a group of individual sample data, the target vital signs among each group of individual sample data have strong correlation and the noise signal has randomness. Combined with Formula (19), the Covariance Matrix of *X_N_*_×*M*_ is:

(21) $$\begin{array}{l} C_{x}=\frac{1}{M}X\cdot X^{T}=\\ \frac{M-1}{M}[\begin{array}{cccc} \mathrm{cov}(\vec{xc_{1}},\vec{xc_{1})} & \mathrm{cov}(\vec{xc_{1}},\vec{xc_{2})} & \cdots & \mathrm{cov}(\vec{xc_{1}},\vec{xc_{N})}\\ \mathrm{cov}(\vec{xc_{2}},\vec{xc_{1})} & \mathrm{cov}(\vec{xc_{2}},\vec{xc_{2})} & \cdots & \mathrm{cov}(\vec{xc_{2}},\vec{xc_{N})}\\ \vdots & \vdots & \ddots & \vdots \\ \mathrm{cov}(\vec{xc_{N}},\vec{xc_{1})} & \mathrm{cov}(\vec{xc_{N}},\vec{xc_{2})} & \cdots & \mathrm{cov}(\vec{xc_{N}},\vec{xc_{N})} \end{array}], \end{array}$$

where cov() represents the covariance between two vectors, the diagonal elements of the covariance matrix *C_x_* represent the variance of the data set and the non-diagonal elements represent the correlation between different dimensions of the data. Because the composition of radar data *X_N_*_×*M*_ is complex and different dimensions of data have strong correlation, the amount of data is large, and some of them cannot be rounded off.

However, according to Equation (19), the following relationship can be obtained:

(22) $$\begin{array}{ll} XX^{T} & =U\Sigma_{1}U^{T}\\ \Rightarrow & U^{T}XX^{T}U=\Sigma_{1}\;,\\ \Rightarrow & U^{T}X{\left(U^{T}X\right)}^{T}=\Sigma_{1} \end{array}$$

Therefore, for matrix *U^T^X*, its covariance matrix is:

(23) $$C_{U^{T}X}=\frac{1}{M}U^{T}X\cdot {\left(U^{T}X\right)}^{T}=\frac{1}{M}\Sigma_{1},$$

Therefore, for radar matrix *X_N_*_×*M*_, after orthogonal similarity transformation by matrix *U^T^*, the data can be projected into a new vector space, where the covariance matrix of the data in the new vector space is a diagonal matrix Σ_1_. The non-diagonal elements of Σ_1_ are zero, indicating that different dimensions are independent of each other in the new data space. The diagonal elements of Σ_1_ are *σ*_1_^2^, *σ*_2_^2^, *σ*_3_^2^, …, *σ_k_*^2^, *σ*_1_^2^ ≥ *σ*_2_^2^ ≥ *σ*_3_^2^ ≥ … ≥ *σ_k_*^2^, indicating that the components corresponding to the fronter elements account for a larger proportion of the data. According to the above characteristics, by selecting the main elements in matrix Σ_1_, one can retain the essential parts and reduce the dimensionality of the original radar data.

Above is the process of dimensionality reduction and denoising for *X_N_*_×*M*_ in the *y*-axis direction. According to the same principle, the covariance matrix of *X^T^* can be analyzed to reduce dimensionality and denoise in *x*-axis direction. The result is the form of SVD as shown in Formula (15).

**Figure 6:**
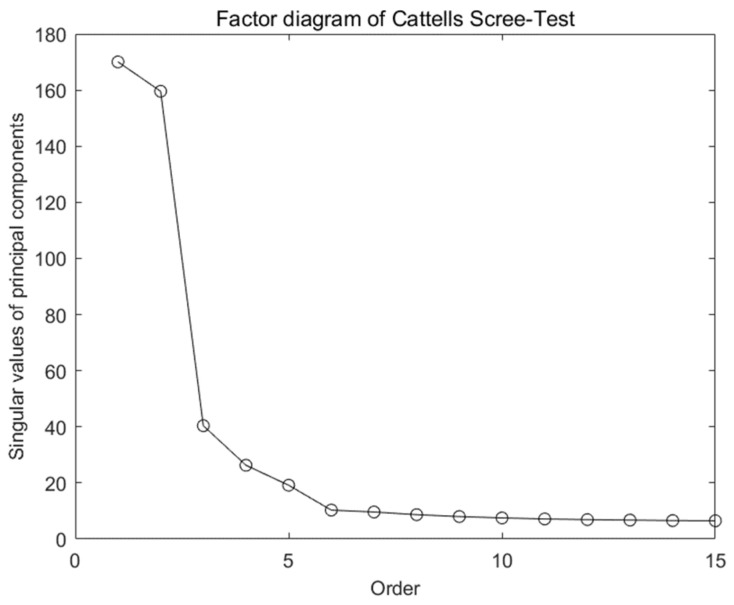
Cattell’s Scree-Test.

For the radar data *X_N_*_×*M*_ after SVD processing, the principal components are screened through Cattell’s Scree-Test. Figure 6 shows the Scree plot of Cattell’s Scree-Test, and the magnitudes and variation trends of major singular values are also shown.

From Figure 6, the second-order to third-order singular values decline rapidly. Combined with Formulas (24) and (25), one can calculate the descent gradient and normalized singular values. After comprehensive consideration, the first six order principal components are selected and retained for subsequent processing after dimensionality reduction and denoising:

(24) $$g_{j}=\frac{\sigma_{j}-\sigma_{j+1}}{\sigma_{1}},\;j=1,2,3,\cdots ,k-1,$$

(25) $$h_{j}=\frac{\sigma_{j}}{\sigma_{1}},\;j=1,2,3,\cdots ,k,$$

**Figure 7:**
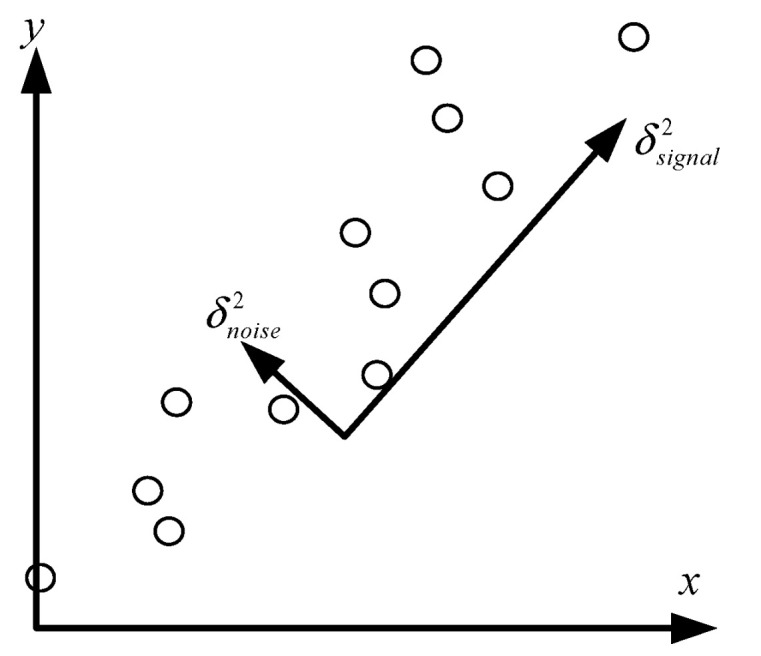
Signal to noise ratio.

In addition, the noise signal *w*[*n*, *m*] has randomness, so its components in each spatial direction are equal. The signal to noise ration sketch map has been shown in Figure 7. The component size of each order principal component is different, but the noise signal contained can be regarded as invariant, so the Signal to Noise Ratio (SNR) of each component can be compared as in Equation (26):

(26) $$SNR(\sigma_{1}u_{1}v_{1})\geq SNR(\sigma_{2}u_{2}v_{2})\geq \cdots \geq SNR(\sigma_{k}u_{k}v_{k}),$$

**Figure 8:**
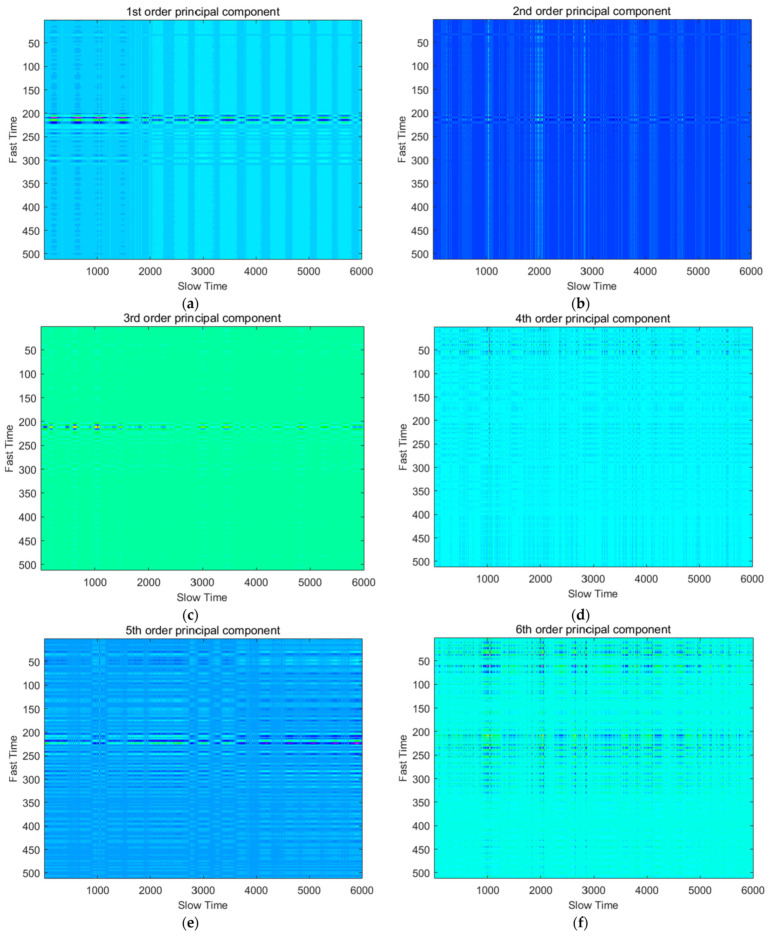
SVD Data denoising and dimensionality reduction effect: (**a**) First order principal component; (**b**) second order principal component; (**c**) third order principal component; (**d**) fourth order principal component; (**e**) fifth order principal component; (**f**) sixth order principal component.

For those small singular values, their corresponding components have a very small signal-to-noise ratio. Therefore, abandoning these components with small signal-to-noise ratio from the original data will cause little loss to the effective components, and at the same time, parts of the noise with relatively large proportions are removed. Figure 8 shows the signal time domain characteristics of the first six order principal components of the radar matrix *X_N_*_×*M*_.

### 5.3. Signal Eigen Decomposition in Time-Space Dimension

After extracting the principal components of the radar matrix, the variety characteristic of each principal component in temporal and spatial dimensions can still be extracted by the SVD algorithm. The principle is as follows:

According to Equations (22) and (23), for radar matrix *X_NxM_*, after orthogonal similarity transformation with *U^T^*, the data are projected into a new space, and each row of matrix *U^T^X* is linearly independent with each other. Noting that *YU* = *U^T^X*, one can obtain:

(27) $$YU=\left[\begin{array}{c} \vec{yu_{1}{}^{T}}\\ \vec{yu_{2}{}^{T}}\\ \vdots \\ \vec{yu_{k}{}^{T}}\\ \vdots \end{array}\right]=\left[\begin{array}{c} \sigma_{1}\cdot \vec{eu_{1}{}^{T}}\\ \sigma_{2}\cdot \vec{\vec{e}u_{2}{}^{T}}\\ \vdots \\ \sigma_{k}\cdot \vec{eu_{k}{}^{T}}\\ \vdots \end{array}\right]=U^{T}X,$$

(28) $$\begin{array}{l} X=U\cdot YU=\left[\vec{u_{1}},\vec{u_{2}},\cdots ,\vec{u_{k}}\right]\left[\begin{array}{c} \sigma_{1}\cdot \vec{eu_{1}{}^{T}}\\ \sigma_{2}\cdot \vec{eu_{2}{}^{T}}\\ \vdots \\ \sigma_{k}\cdot \vec{eu_{k}{}^{T}} \end{array}\right]\\ =\sigma_{1}\vec{u_{1}}\vec{eu_{1}{}^{T}}+\sigma_{2}\vec{u_{2}}\vec{eu_{2}{}^{T}}+\cdots +\sigma_{k}\vec{u_{k}}\vec{eu_{k}{}^{T}} \end{array}$$

where the vectors *eu*_1_, *eu*_2_, …, *eu_k_* are the unit orthogonal bases corresponding to each order principal components in the *x*-axis direction, which are linearly independent of each other. The left singular matrix *U* projects and restores each order principal components which feature in the *x*-axis direction to the original radar matrix *X_N_*_×*M*_. It can be derived that each order left singular vectors *u_i_* reflect the variation characteristics of the principal components along the *y*-axis, namely, *u_i_* decomposes the variation characteristics of the radar matrix *X_N_*_×*M*_ in spatial dimension. In this paper, *u_i_* is noted as the *i*th spatial eigenvector of the radar matrix *X_N_*_×*M*_. By analyzing *u_i_*, one can obtain the variation characteristics of radar signal components at different detection distance.

Similarly, noting that *YV* = *V^T^X*, one can obtain:

(29) $$YV=\left[\begin{array}{c} \vec{yv_{1}{}^{T}}\\ \vec{yv_{2}{}^{T}}\\ \vdots \\ \vec{yv_{k}{}^{T}}\\ \vdots \end{array}\right]=\left[\begin{array}{c} \sigma_{1}\cdot \vec{ev_{1}{}^{T}}\\ \sigma_{2}\cdot \vec{\vec{e}v_{2}{}^{T}}\\ \vdots \\ \sigma_{k}\cdot \vec{ev_{k}{}^{T}}\\ \vdots \end{array}\right]=V^{T}X^{T},$$

(30) $$\begin{array}{l} X^{T}=V\cdot YV=\left[\vec{v_{1}},\vec{v_{2}},\cdots ,\vec{v_{k}}\right]\left[\begin{array}{c} \sigma_{1}\cdot \vec{ev_{1}{}^{T}}\\ \sigma_{2}\cdot \vec{ev_{2}{}^{T}}\\ \vdots \\ \sigma_{k}\cdot \vec{ev_{k}{}^{T}} \end{array}\right]\\ =\sigma_{1}\vec{v_{1}}\vec{ev_{1}{}^{T}}+\sigma_{2}\vec{v_{2}}\vec{ev_{2}{}^{T}}+\cdots +\sigma_{k}\vec{v_{k}}\vec{ev_{k}{}^{T}} \end{array}$$

**Figure 9:**
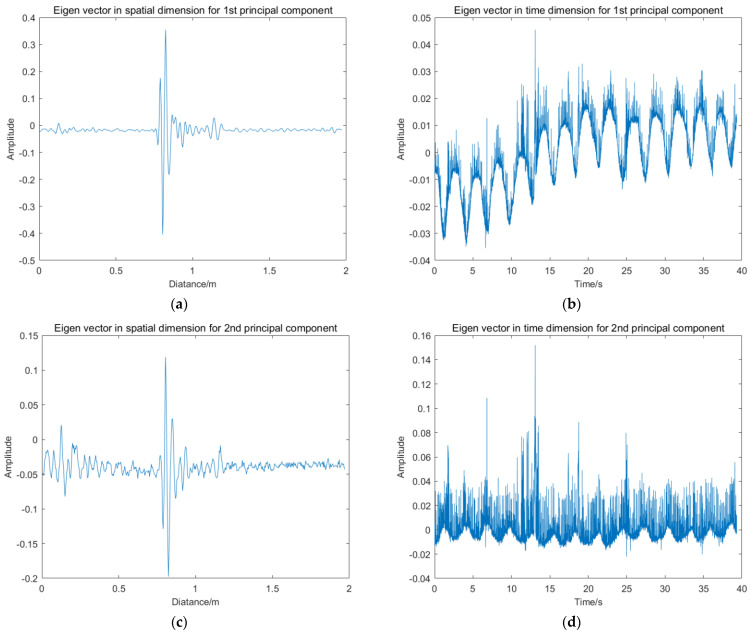

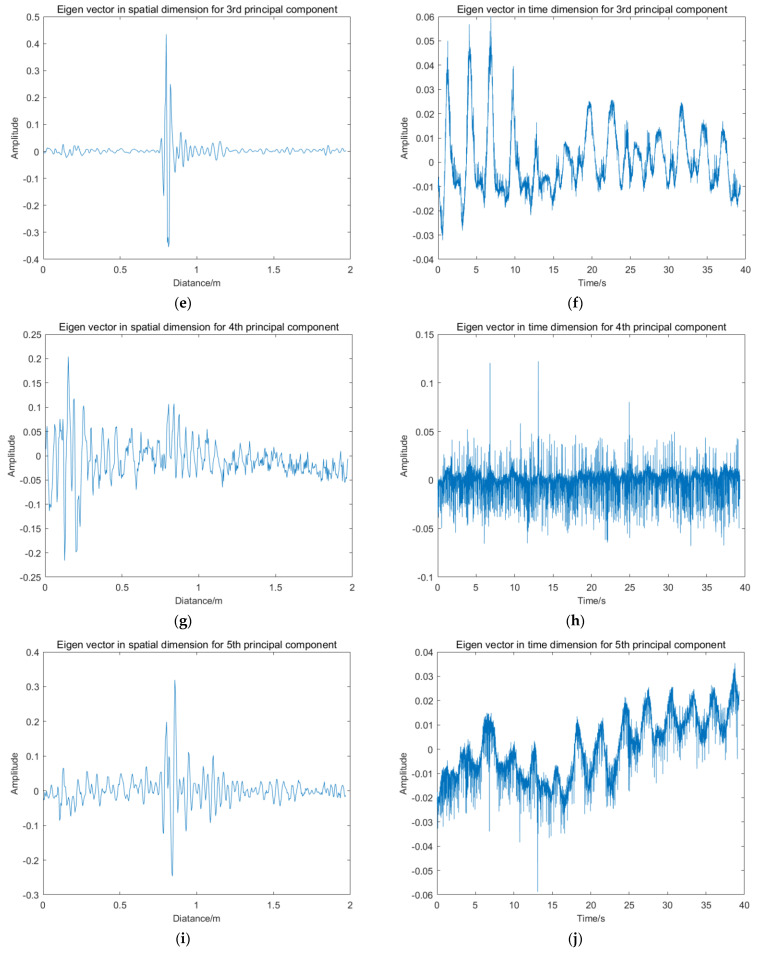

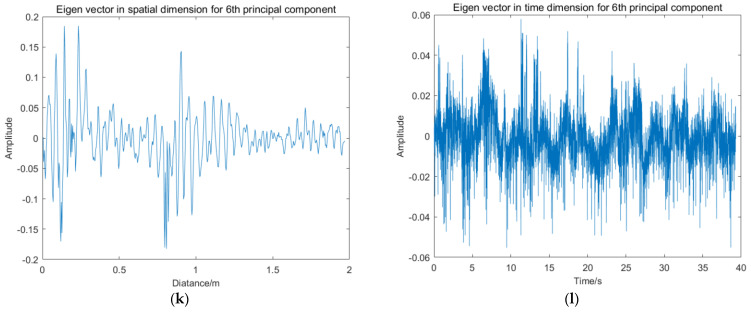
Temporal and spatial eigenvectors for the first six order principal components: (**a**) First spatial eigenvector; (**b**) first temporal eigenvector; (**c**) second spatial eigenvector; (**d**) second temporal eigenvector; (**e**) third spatial eigenvector; (**f**) third temporal eigenvector; (**g**) fourth spatial eigenvector; (**h**) fourth temporal eigenvector; (**i**) fifth spatial eigenvector; (**j**) fifth temporal eigenvector; (**k**) sixth spatial eigenvector; (**l**) sixth temporal eigenvector.

Each order right singular vectors *v_i_* reflect the variation characteristics of the principal components along the *x*-axis, namely, *v_i_* decomposes the variation characteristics of the radar matrix *X_N_*_×*M*_ in the temporal dimension. In this paper, *v_i_* is noted as the *i*th temporal eigenvector of the radar matrix *X_N_*_×*M*_. By analyzing *v_i_*, one can obtain the variation characteristics of radar signal components at different detection time. Figure 9 shows the temporal and spatial eigenvectors for the first six order principal components.

## 6. Vital Signs Extraction and Restoration

### 6.1. Signal Components Screening Based on Energy Proportion Analysis

**Figure 10:**
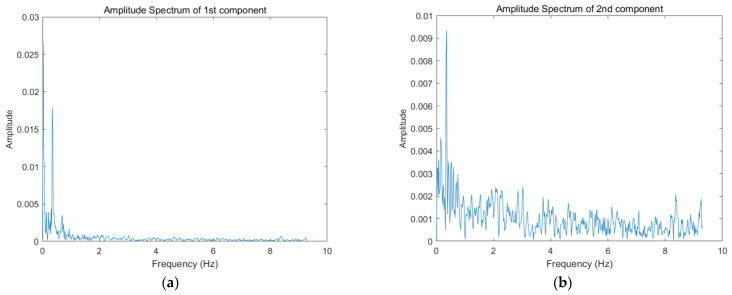

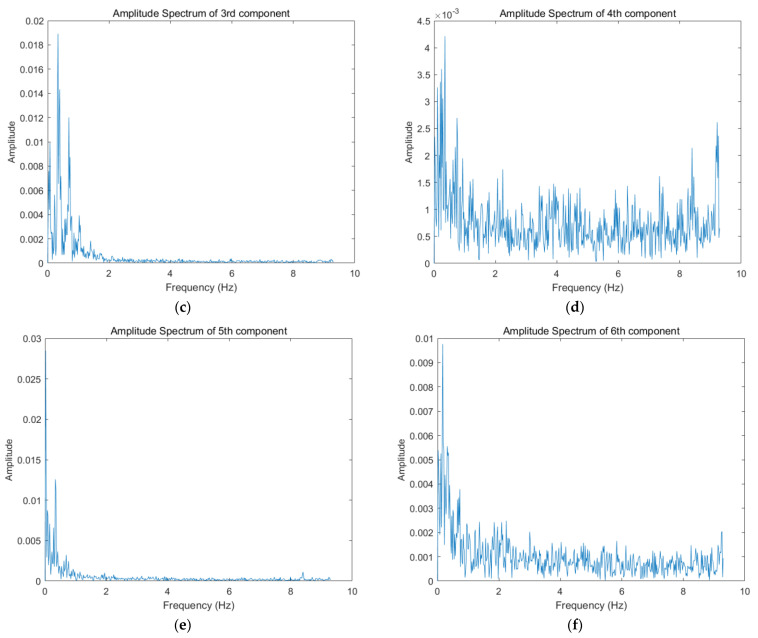
Amplitude spectrum of each order principal component: (**a**) Amplitude spectrum of first component; (**b**) Amplitude spectrum of second component; (**c**) Amplitude spectrum of third component; (**d**) Amplitude spectrum of fourth component; (**e**) Amplitude spectrum of fifth component; (**f**) Amplitude spectrum of sixth component.

**Figure 11:**
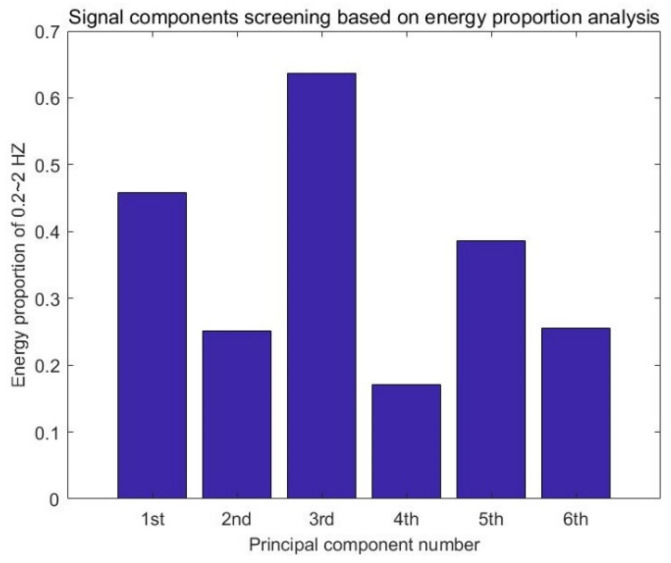
Energy proportion diagram of vital signs frequency band.

For each order principal component extracted by SVD, some clutters and noise still exist. For the principal components with much noise and only a small number of vital signs, it is difficult to extract effective information because of the low signal-to-noise ratio, therefore, it is not necessary to perform further analysis. For screening, these signals are first converted to the frequency domain by fast Fourier transform. Generally, the frequency range of human cardiopulmonary movements such as respiration and heartbeats are 0.2 to 0.7 Hz and 1.1 to 2 Hz, respectively. Therefore, the range of frequency domain characteristic extracted after fast Fourier transform is set between 0.2 and 2 Hz to cover the frequency range of the whole vital sign signal. Figure 10 shows the amplitude spectrum of each order principal component. By calculating the ratio of the vital signs’ frequency band energy to the whole signal frequency band energy, one can obtain the energy proportion diagram of vital signs frequency band shown in Figure 11. Finally, the first, third and fifth order principal components were selected for further analysis and processing.

### 6.2. Vital Signs Extraction Based on Wavelet Transformation

In case of practical engineering applications, the analyzed signal may contain many spikes or abrupt parts, and the noise is not always stationary white noise. In this case, the traditional Fourier analysis is incapable of action because it cannot reflect the signal change at a certain time point, so that any sudden change in the time axis will affect the whole spectrum of the signal. The wavelet analysis can carry out multi-resolution analysis on the signal in time and frequency domain at the same time, so it can effectively distinguish the abrupt part and noise in the signal, so as to realize signal denoising.

Assume that the received signal has the following form:

(31) f(t)=s(t)+u(t),

where *s*(*t*) is the target signal without noise, *u*(*t*)~*N*(0, *σ*^2^) is the steady additive Gaussian white noise with the mean value of zero and variance of *σ^2^*. By discrete sampling of the signal *f*(*t*), one can obtain:

(32) $$f(n),\begin{array}{cc} & n=0,1,\ldots ,N-1 \end{array},$$

The wavelet transform process is:

(33) $$WT_{f}(j,k)=2^{-\frac{j}{2}}\sum_{n=0}^{N-1}f(n)\psi (2^{-j}n-k),$$

where *WT_f_* (*j*, *k*) is the wavelet coefficient, and *j* represents the number of wavelet decomposition layers. In practical application, in order to simplify the process, the recursive implementation method of wavelet transform is always obtained according to the double-scale equation, namely, the Mallet algorithm:

(34) $$\begin{array}{l} S_{f}(0,k)=f(k)\\ S_{f}(j+1,k)=S_{f}(j,k)*h(j,k)\\ WT_{f}(j+1,k)=S_{f}(j,k)*g(j,k) \end{array}$$

where *h* and *g* are low-pass and high-pass filters corresponding to scale function *δ*(*x*) and wavelet function *ψ*(*x*), respectively, *S_f_* (*j*, *k*) is scale coefficient and *WT_f_* (*j*, *k*) is the wavelet coefficient. Accordingly, the reconstruction formula of the wavelet transform is:

(35) $$S_{f}(j-1,k)=S_{f}(j,k)*\tilde{h}(j,k)+WT_{f}(j,k)*\tilde{g}(j,k),$$

**Figure 12:**
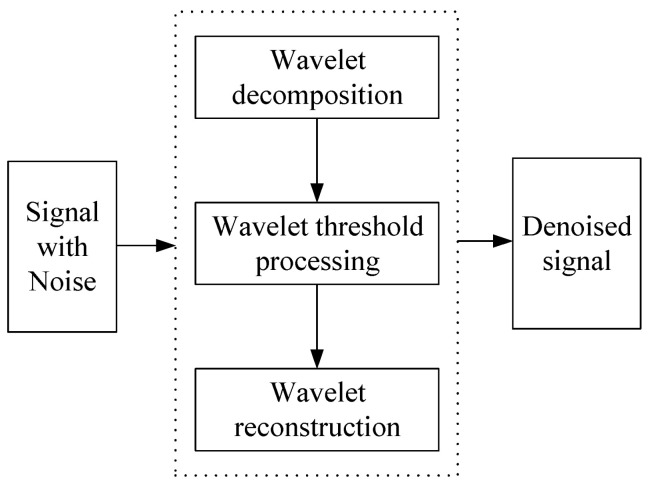
Wavelet transformation signal denoising process.

The process of signal denoising by wavelet transform is shown in Figure 12.

Perform wavelet transform on the noisy signal of Equation (31), noting *W* as the wavelet transform matrix and *f* and *s* as the vectors corresponding to *f*(*n*) and *s*(*n*), respectively. Then, one can obtain:

(36) $$WT_{f}=Wf,WT_{s}=Ws,WT_{u}=Wu,$$

According to the linear property of wavelet transform, one can obtain:

(37) $$WT_{f}=WT_{s}+WT_{u},$$

According to the property of noise *u*, there are:

(38) $$\begin{array}{l} E\left\{WT_{u}\right\}=E\{Wu\}=WE\{u\}=0\\ P\{WT_{u}\}=E\left\{WT_{u}WT_{u}{}^{T}\right\}=E\left\{Wuu^{T}W^{T}\right\}=\sigma^{2}I \end{array},$$

According to the above properties, after orthogonal wavelet transformation, the energy of wavelet coefficients *WT_f_* (*j*, *k*) on each scale corresponding to signal *s*(*n*) is mainly concentrated in a few specific positions, which correspond to the odd positions and important information of original signal *s*(*n*). The noise *u*(*n*) is still white noise after orthogonal wavelet transformation, and its wavelet coefficients are uncorrelated to each other and distributed on all time axes at all scales.

Based on above characteristic, the process and principle of wavelet denoising are as follows: First, wavelet transformation is applied to the noisy signal. Then, the wavelet coefficients of the target signal are extracted by various methods at each scale. At the same time, the wavelet coefficients of noise are removed as much as possible. Finally, the signal is reconstructed by inverse wavelet transformation to achieve the purpose of denoising.

In this paper, the *sym6* wavelet base is used. The commonly used threshold functions are hard threshold function and soft threshold functions, which are given by Formulas (39) and (40), respectively:

(39) $${\hat{w}}_{j,k}=\{\begin{array}{ccc} w_{j,k} & & \left|w_{j,k}\right|\geq \lambda \\ 0 & & \left|w_{j,k}\right|<\lambda \end{array},$$

(40) $${\hat{w}}_{j,k}=\{\begin{array}{ccc} \mathrm{sgn}(w_{j,k})(\left|w_{j,k}\right|-\lambda ) & & \left|w_{j,k}\right|\geq \lambda \\ 0 & & \left|w_{j,k}\right|<\lambda \end{array},$$

The hard threshold function can retain the peak characteristics of the original signal as much as possible, while the soft threshold function has good continuity at the threshold boundary. Therefore, the hard threshold function is used to denoise the spatial eigenvectors *u_i_* in the spatial dimension, and the soft threshold function is used to denoise the temporal eigenvectors *v_i_* in the time dimension.

**Figure 13:**
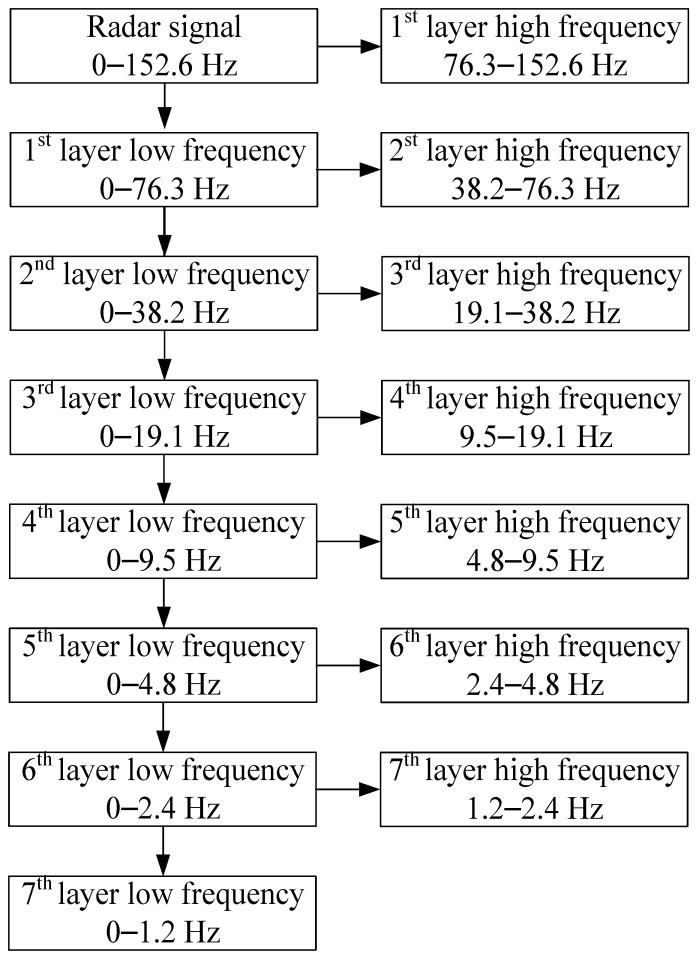
Frequency range of radar signal under wavelet multi-scale decomposition.

**Figure 14:**
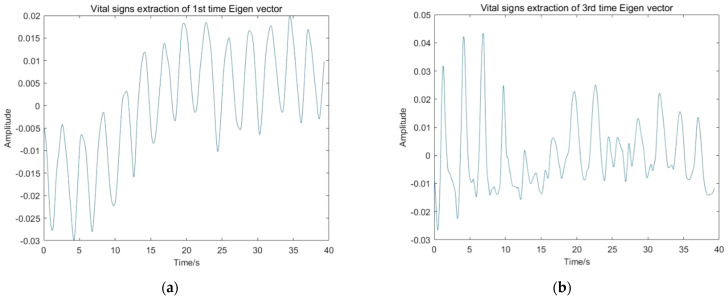

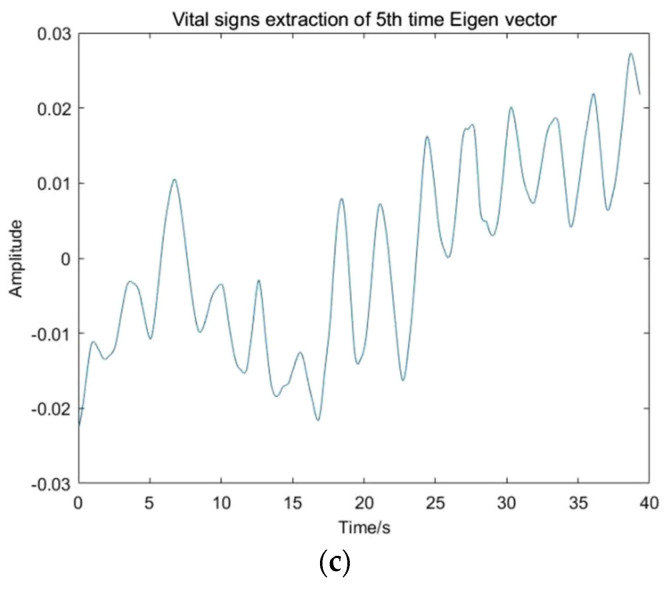
Vital signs extraction results of each principal component: (**a**) Vital signs extraction of first temporal eigenvector; (**b**) Vital signs extraction of second temporal eigenvector; (**c**) Vital signs extraction of third temporal eigenvector.

In this paper, the frequency of radar pulse transmission is 152.6 Hz, while the frequency range of vital signs is 0.2–2 Hz. Therefore, when extracting vital signs from the temporal eigenvectors *v_i_*, the number of wavelet decomposition layers is set to seven. The frequency range of the radar signal under wavelet multi-scale decomposition is shown in Figure 13. The threshold of noise reduction is determined with the penalty strategy. According to the frequency range, the energy of vital signs is concentrated on the seventh layer wavelet component, so only the seventh layer low-frequency and high-frequency components are retained for further denoising to extract vital signs. The vital signs extraction results of each principal component are shown in Figure 14.

### 6.3. Survivor Location Based on Energy Entropy

**Figure 15:**
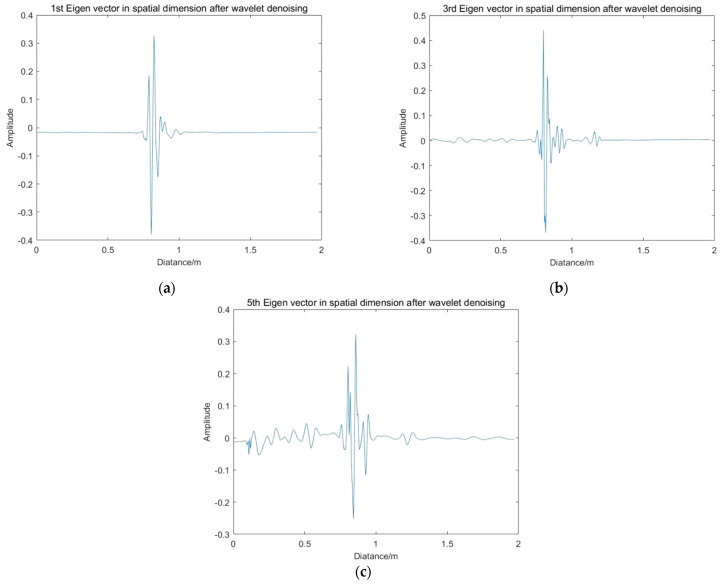
Spatial eigenvectors after wavelet denoising: (**a**) First spatial eigenvector after wavelet denoising; (**b**) third spatial eigenvector after wavelet denoising; (**c**) fifth spatial eigenvector after wavelet denoising.

As described in the previous section, wavelet transform is used to denoise the spatial eigenvector. The results of wavelet denoising of *u_i_* by using the hard threshold function are shown in Figure 15.

**Figure 16:**
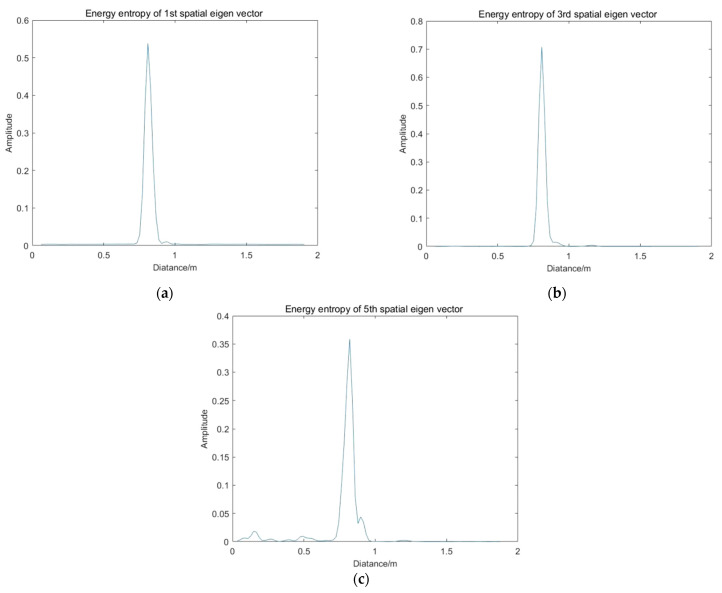
Energy entropy of each order principal component at different positions: (**a**) Energy entropy of first spatial eigenvector; (**b**) Energy entropy of third spatial eigenvector; (**c**) Energy entropy of fifth spatial eigenvector.

Then, constructing the Hamming window according to the thickness of human chest, calculate the energy entropy of each order principal component at different positions. The results are shown in Figure 16. As shown in Table 1, the maximum energy entropy is the location of the target vital signs. At the same time, the confidence factors (*K*) are calculated according to the signal-to-noise ratio of each order spatial eigenvector.

Different proportions are given according to the singular value and signal-to-noise ratio of each order principal component, the final calculated survivor target position is *P_c_* = 0.811 m. Therefore, the positioning error of the algorithm proposed in this paper is *err* = 0.011 m.

### 6.4. Respiratory Signal Restoration

The vital signs extracted from each order principal components shown in Figure 14 have removed a large amount of high-frequency noise. Therefore, except for the vital signs of respiration and heartbeats, the residual clutter in the current signal is the low-frequency linear drift. The principle of linear trend suppression is as follows:

(41) $$W=\Omega^{T}-X{\left(X^{T}X\right)}^{-1}X^{T}\Omega^{T},$$

**Figure 17:**
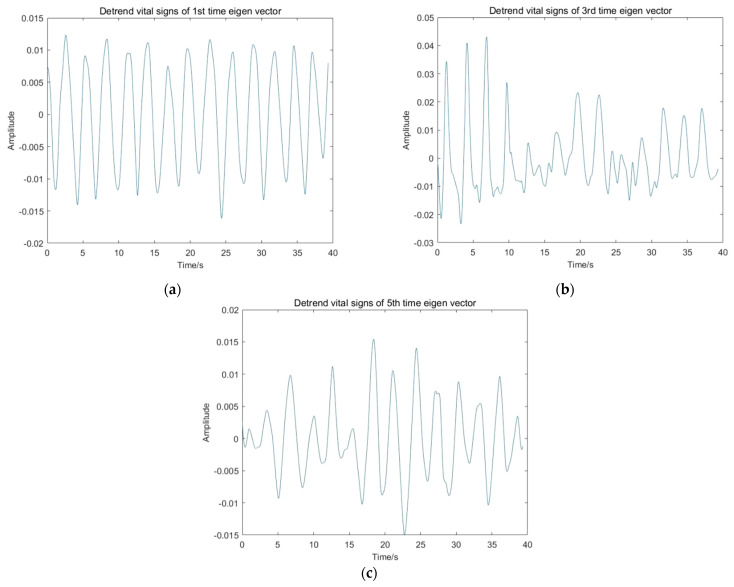
Linear trend suppression effects of vital signs: (**a**) Detrend vital signs of first temporal eigenvector; (**b**) Detrend vital signs of third temporal eigenvector; (**c**) Detrend vital signs of fifth temporal eigenvector.

The linear trend suppression effects are shown in Figure 17.

**Figure 18:**
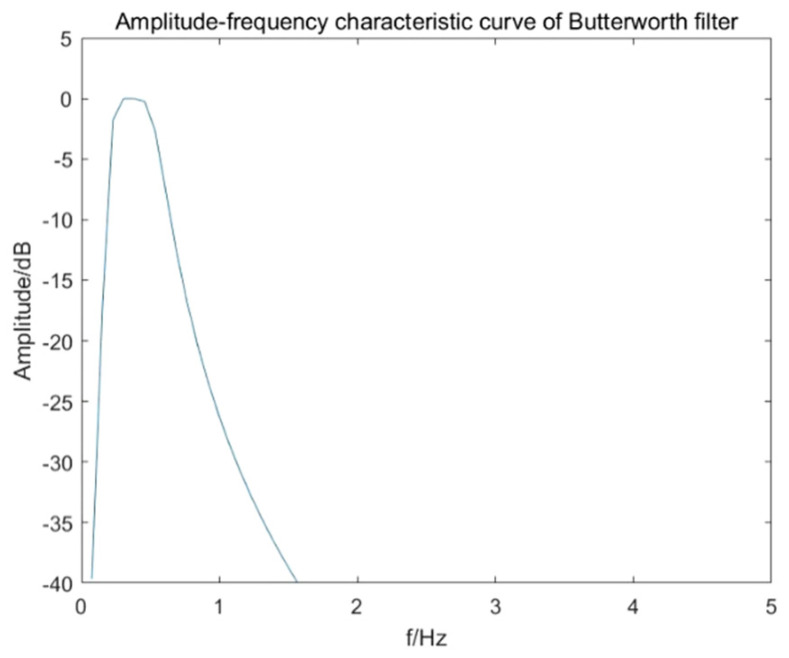
Amplitude-frequency characteristic of Butterworth filter for respiratory signal extraction.

In order to extract the target respiratory signal of 0.2–0.6 Hz in vital signs, the Butterworth filter is adopted according to the properties of original signal and target signal. The Butterworth filter is an infinite impulse response (IIR) digital filter, which is characterized by a frequency response curve that is maximally flat in the passband without ripple and gradually drops to zero in the stopband. In order to extract the breathing signal, a Butterworth bandpass filter needs to be designed, and its transfer function can be expressed as:

(42) $$H(u,v)=\frac{1}{1+{\left[\frac{D^{2}(u,v)-D_{0}{}^{2}}{D(u,v)\cdot W}\right]}^{2n}},$$

where *D*_0_ is the center frequency of the passband, *W* is the bandwidth of the bandpass filter and *n* is the order. In order to obtain a purer signal and ensure that the filter can effectively extract the signal, the upper and lower boundary frequencies of the passband are set to 0.2 Hz and 0.6 Hz, respectively, the passband attenuation is set to 0.1 dB. In addition, the upper and lower boundary frequencies of the stopband are set to 0.05 Hz and 0.8 Hz, the stopband attenuation is set to 1 dB. The order of the Butterworth filter *n* is calculated equals three, and its amplitude-frequency characteristic curve is shown in Figure 18.

**Figure 19:**
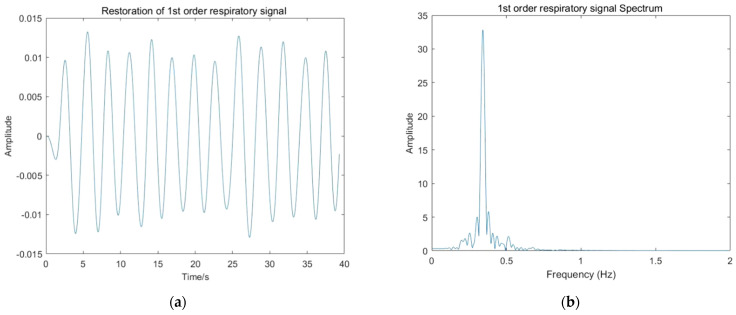

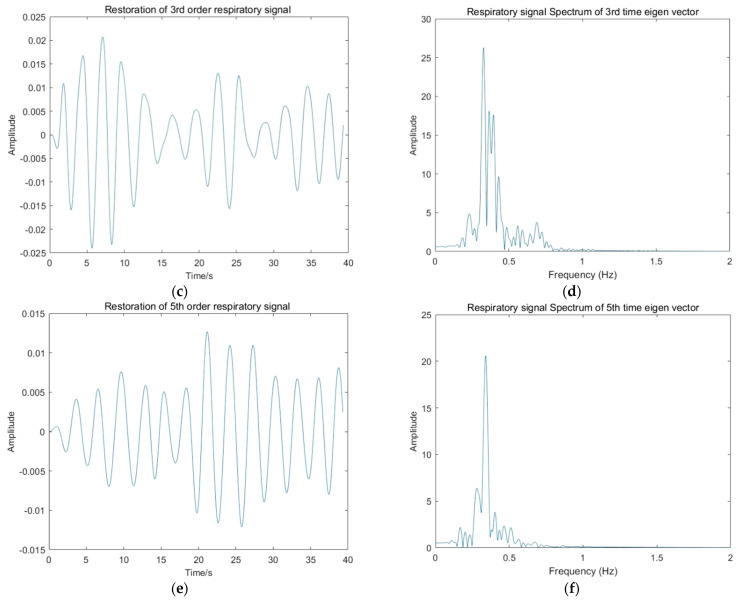
Respiratory signal restoration result: (**a**) Restoration of first order respiratory signal; (**b**) first order respiratory signal spectrum; (**c**) Restoration of third order respiratory signal; (**d**) third order respiratory signal spectrum; (**e**) Restoration of fifth order respiratory signal; (**f**) fifth order respiratory signal spectrum.

The restored respiratory signals and their spectra are shown in Figure 19.

Extracting the maximum value in each order spectrum as the respiratory signal frequency and calculating the signal-to-noise ratio according to the following equation, one can obtain the results shown in Table 2:

(43) $$\begin{array}{ll} SNR & =10\times \log10\frac{sigPower}{noisePower}\\ & =10\times \log10\frac{\sum_{f=RF-0.025}^{RF+0.025}Ak(f)}{sum(Ak(f))-\sum_{f=RF-0.025}^{RF+0.025}Ak(f)} \end{array},$$

### 6.5. Heartbeat Signal Restoration

Among the extracted principal components of each order, the heartbeat signal component is only observed in the spectrum of the third temporal eigenvector. However, because of the weak strength, it is still difficult to be directly visible, even after clutter and noise removal performance.

**Figure 20:**
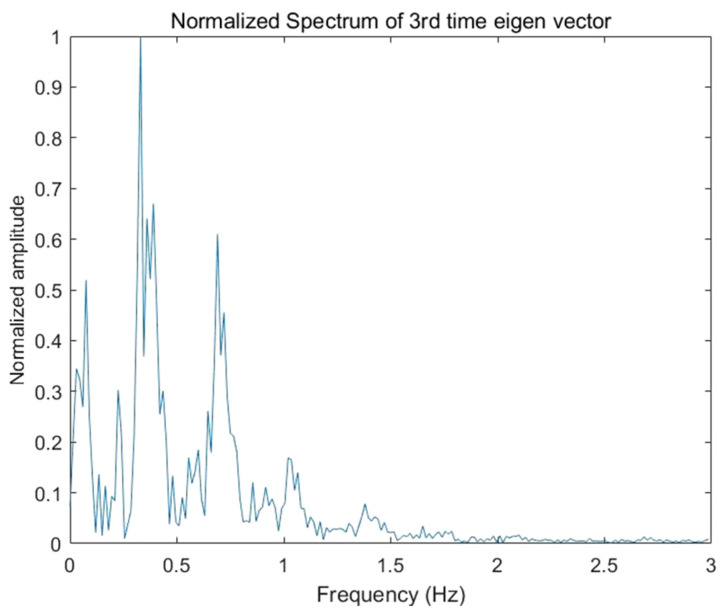
Normalized spectrum of the third temporal eigenvector.

Figure 20 shows the normalized spectrum of the third temporal eigenvector, the position of the maximum peak corresponds to the respiratory frequency, and the positions of the second and third peaks correspond to two and three times of the respiratory frequency. Therefore, it can be seen that the high-order harmonics of respiratory signal produce great interference.

**Figure 21:**
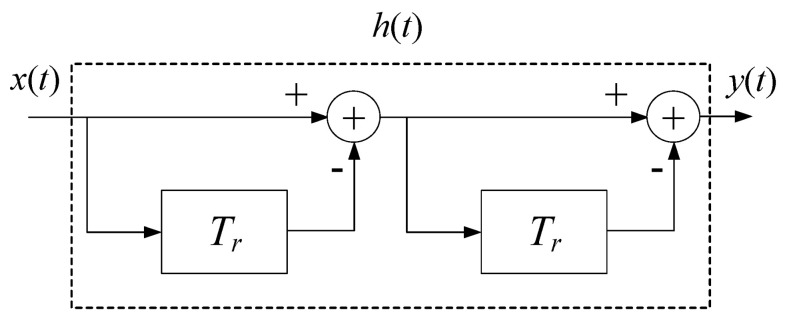
The principle of a double-delay MTI harmonic canceller.

The Moving Target Indicator (MTI) method is adopted to attenuate the breathing harmonics. Figure 21 shows the principle of a double-delay MTI harmonic canceller. Set the system delay *T_r_* to the inverse of the respiratory frequency *RF*. The response of the double-delay MTI harmonic canceller system is demonstrated in Equation (45):

(44) $$T_{r}=\frac{1}{RF},$$

(45) $$h(t)=\delta (t)-2\delta (t-T_{r})+\delta (t-2T_{r}),$$

The transfer function of the system is as follows:

(46) $$H(\omega )={\left(1-e^{-j\omega T_{r}}\right)}^{2}={\left(2j\sin(\omega T_{r})\right)}^{2}e^{-j\omega T_{r}},$$

According to the above system transfer equation, it is easy to infer that the filter can suppress the signal frequencies that are multiples of respiratory frequency *RF*. One can obtain a good effect of the MTI harmonic canceller processing on the radar signal.

**Figure 22:**
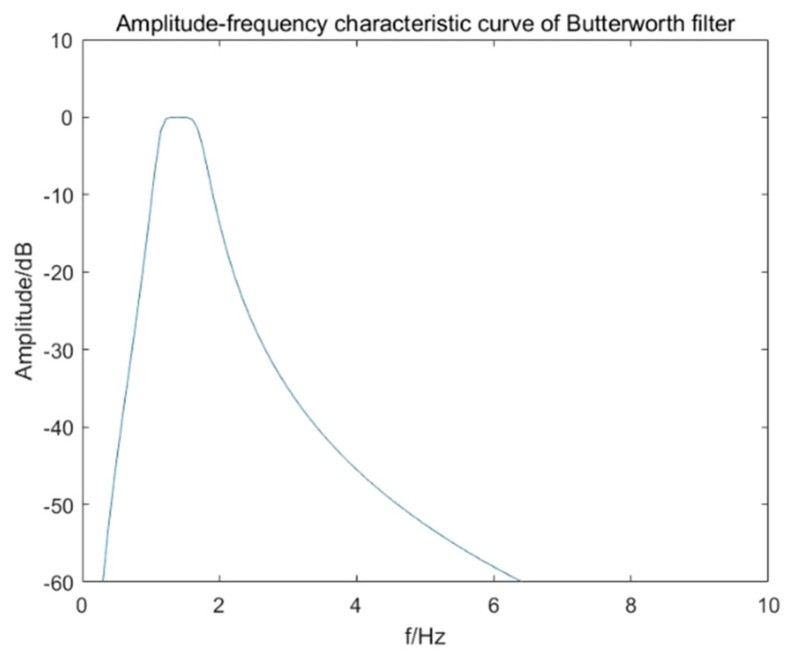
Amplitude–frequency characteristic of Butterworth filter for heartbeat signal extraction.

Same as extracting the respiratory signal, build a Butterworth filter to extract the heartbeat signal from the harmonic cancelled signal. The upper and lower boundary frequencies of the passband are set to 1.0 Hz and 1.9 Hz respectively, the passband attenuation is set to 0.1 dB. The upper and lower boundary frequencies of the stopband are set to 0.7 Hz and 2.2 Hz, respectively, the stopband attenuation is set to 0.6 dB. The order of the Butterworth filter n equals 3, and its amplitude–frequency characteristic curve is shown in Figure 22.

**Figure 23:**
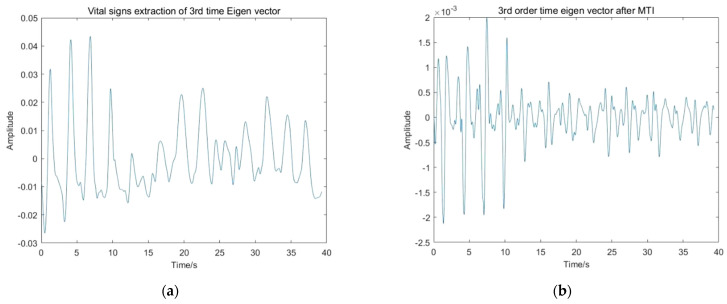

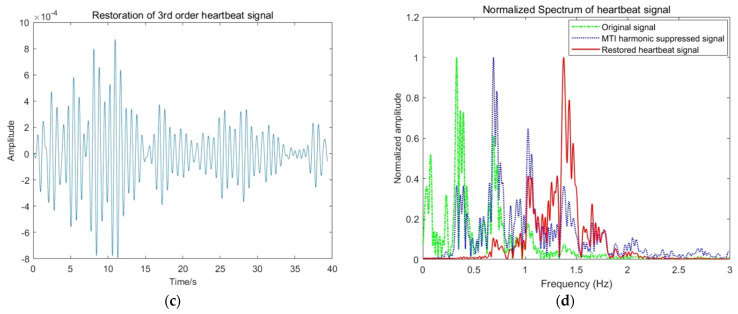
Heartbeat signal restoration result: (**a**) Original signal; (**b**) MTI harmonic suppressed signal; (**c**) Restored heartbeat signal; (**d**) Normalized spectrum.

The restoration results and normalized spectrum of the original signal and each step’s processed signal are shown in Figure 23c,d.

Extracting the maximum value in the spectrum as the heartbeat signal frequency, and calculating the signal-to-noise ratio, one can obtain the results shown in Table 3.

### 6.6. Vital Signs Reconstruction and Result Analysis

**Figure 24:**
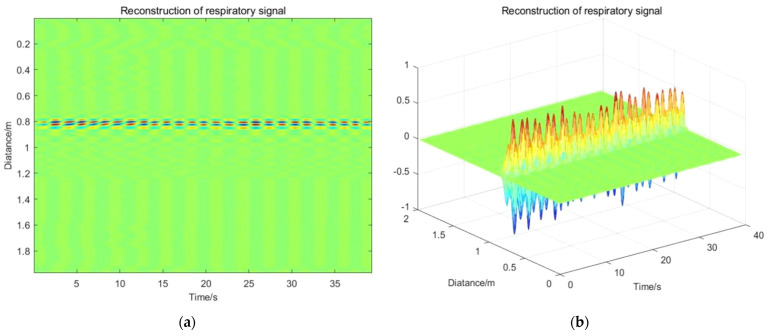

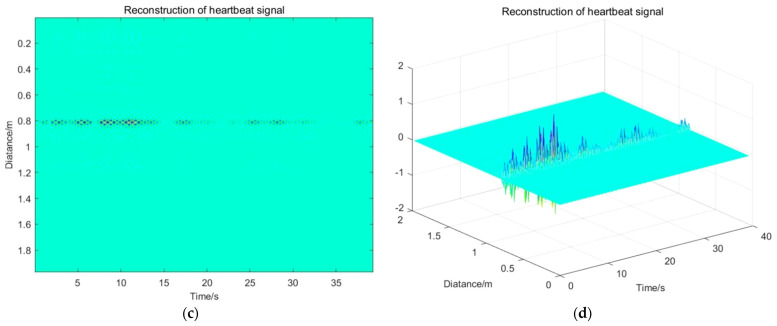
Vital signs reconstruction results: (**a**) Reconstruction of respiratory signal; (**b**) Three-dimensional display of respiratory signal; (**c**) Reconstruction of heartbeat signal; (**d**) Three-dimensional display of heartbeat signal.

According to the above research works, the vital sign reconstruction results are shown in Figure 24. The performance of the proposed algorithm in this paper is shown in Table 4.

During the experimental tests, the vital signs are detected with the UWB radar life detector, meanwhile and the respiratory and heartbeat frequencies of the target human body are measured. The target human body breathes 22 times per minute and their heart beats 76 times per minute. The true values of the respiratory frequency and heartbeat frequency are 0.367 Hz and 1.267 Hz, respectively. For the above simple scenario, the running time of the algorithm on a specific experiment platform is 2.18 s. The above results show the good properties of the algorithm.

## 7. Performance Verification and Comparison

In order to verify the effectiveness of the algorithm proposed in this paper, five volunteers were chosen for the validation experiment; A, B, C, D are healthy adult males (A: 26 years old, 176 cm, 72 kg; B: 30 years old, 183 cm, 96 kg; C: 38 years old, 174 cm, 63 kg; D: 42 years old, 171 cm, 86 kg); E is a healthy adult female (E: 23 years old, 160 cm, 48 kg). The results are shown in Table 5.

The above results show that the proposed algorithm has good effectiveness, accuracy and efficiency under simple detection conditions. For a demonstration of the performance of the proposed method in complex environment, several experiments under different conditions have been executed. During the experiments, different detection distances, different personnel numbers and characteristics, indoor and outdoor environment, whether there are obstacles, etc., are used for the construction of different detection conditions. The basic experimental platform architectures have been shown in Figure 3. The settings of different experimental control groups are shown in Table 6. It is worth noting that the dynamic target shown in the table does not mean that the tested person is moving in a large range but refers to his small-scale activities such as stepping. In addition, in order to achieve long-distance detection, the fast time sampling frequency of the UWB radar system is changed to 5 Ghz, and the slow time impulse frequency is changed to 10 Hz.

**Figure 25:**
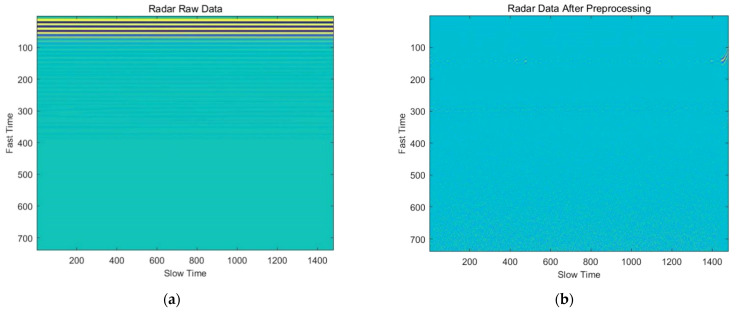

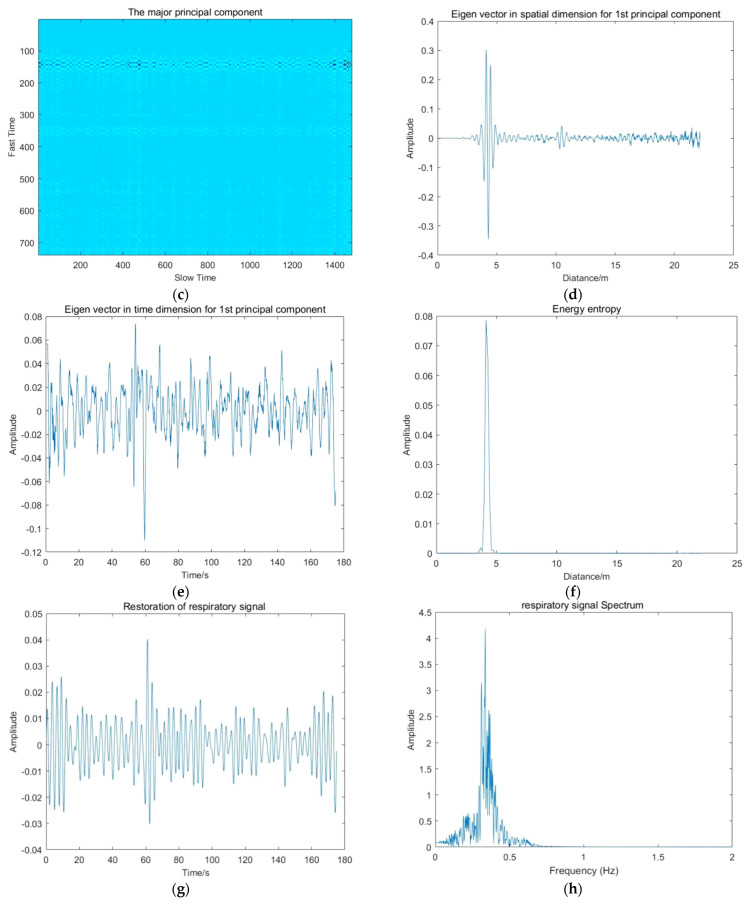

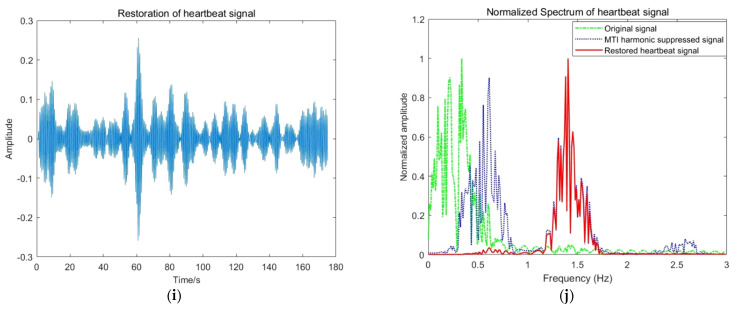
Results of essential steps for experiment group 2: (**a**) Radar raw data; (**b**) Radar data after preprocessing; (**c**) The relative principal component; (**d**) Spatial eigenvector; (**e**) Temporal eigenvector; (**f**) Energy entropy; (**g**) Restoration of respiratory signal; (**h**) Respiratory signal spectrum; (**i**) Restoration of heartbeat signal; (**j**) Heartbeat signal spectrum.

**Figure 26:**
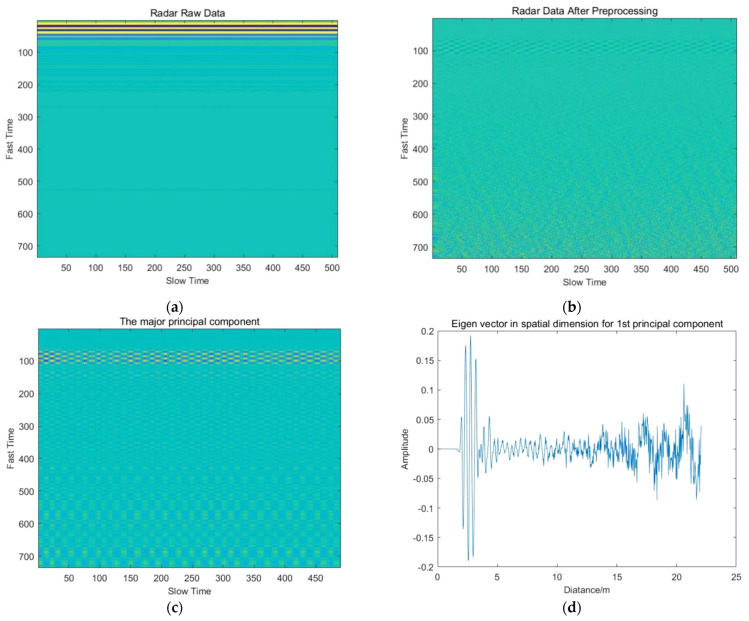

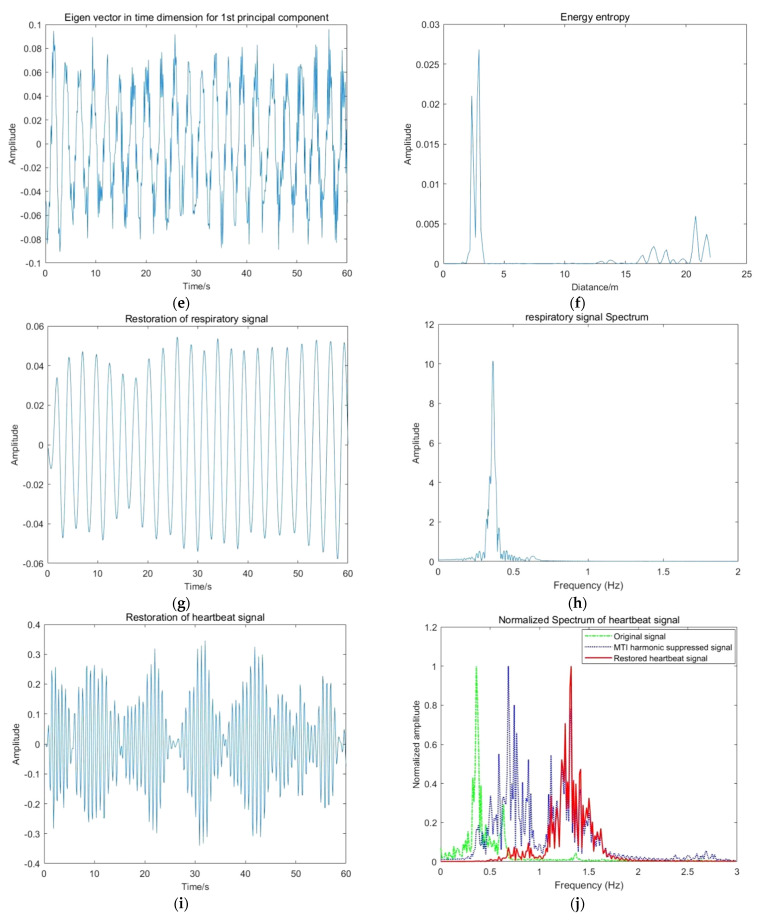
Results of essential steps for experiment group 3: (**a**) Radar raw data; (**b**) Radar data after preprocessing; (**c**) The relative principal component; (**d**) Spatial eigenvector; (**e**) Temporal eigenvector; (**f**) Energy entropy; (**g**) Restoration of respiratory signal; (**h**) Respiratory signal spectrum; (**i**) Restoration of heartbeat signal; (**j**) Heartbeat signal spectrum.

**Figure 27:**
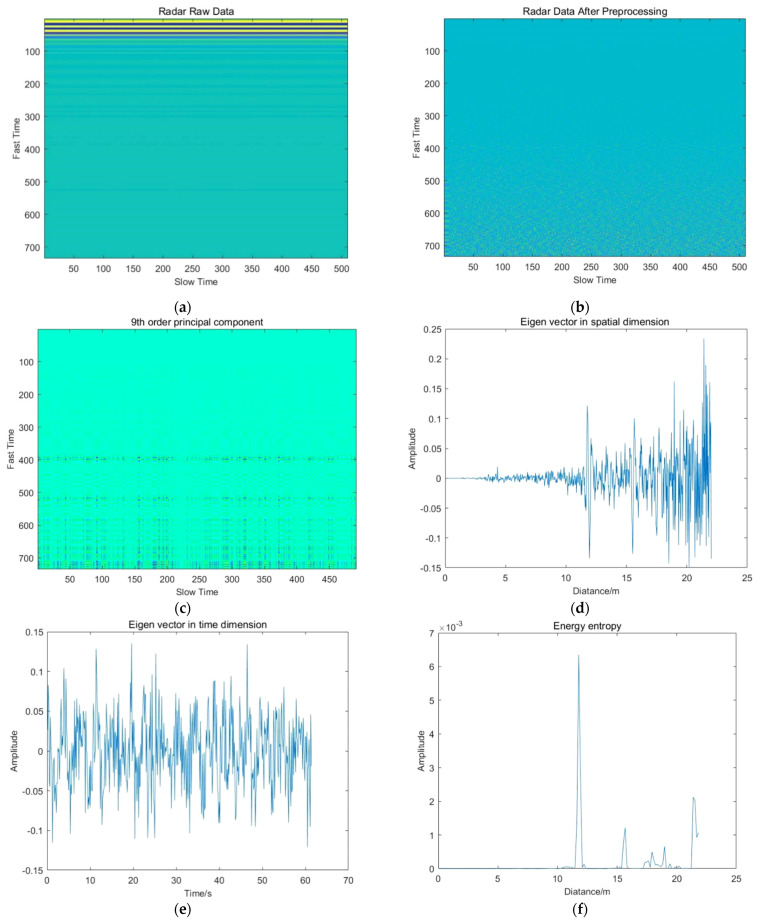

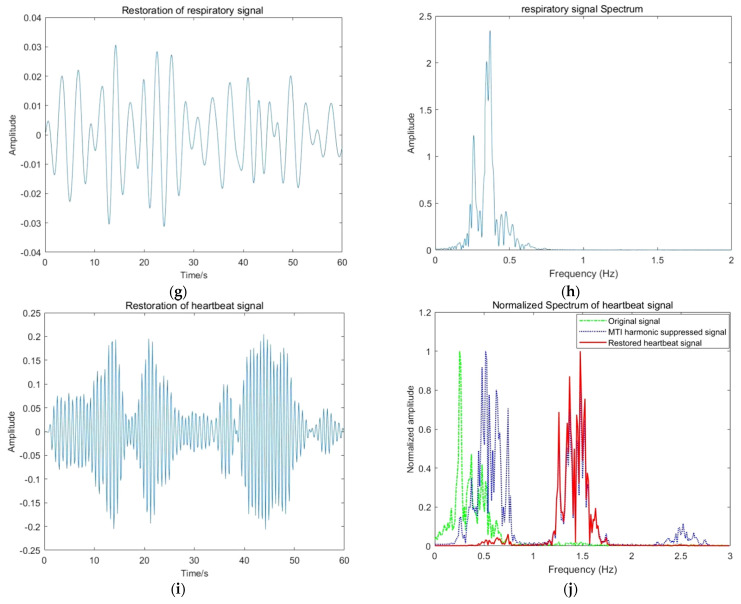
Results of essential steps for experiment group 4: (**a**) Radar raw data; (**b**) Radar data after preprocessing; (**c**) The relative principal component; (**d**) Spatial eigenvector; (**e**) Temporal eigenvector; (**f**) Energy entropy; (**g**) Restoration of respiratory signal; (**h**) Respiratory signal spectrum; (**i**) Restoration of heartbeat signal; (**j**) Heartbeat signal spectrum.

**Figure 28:**
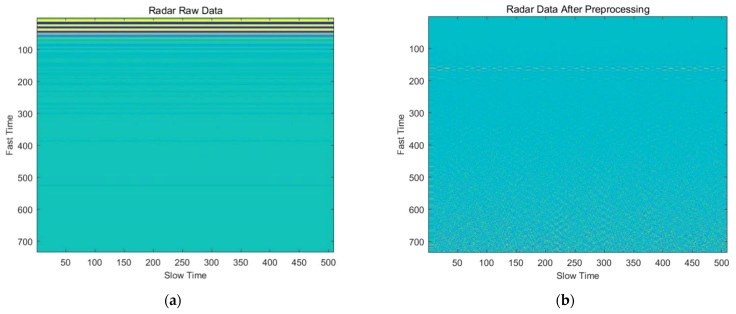

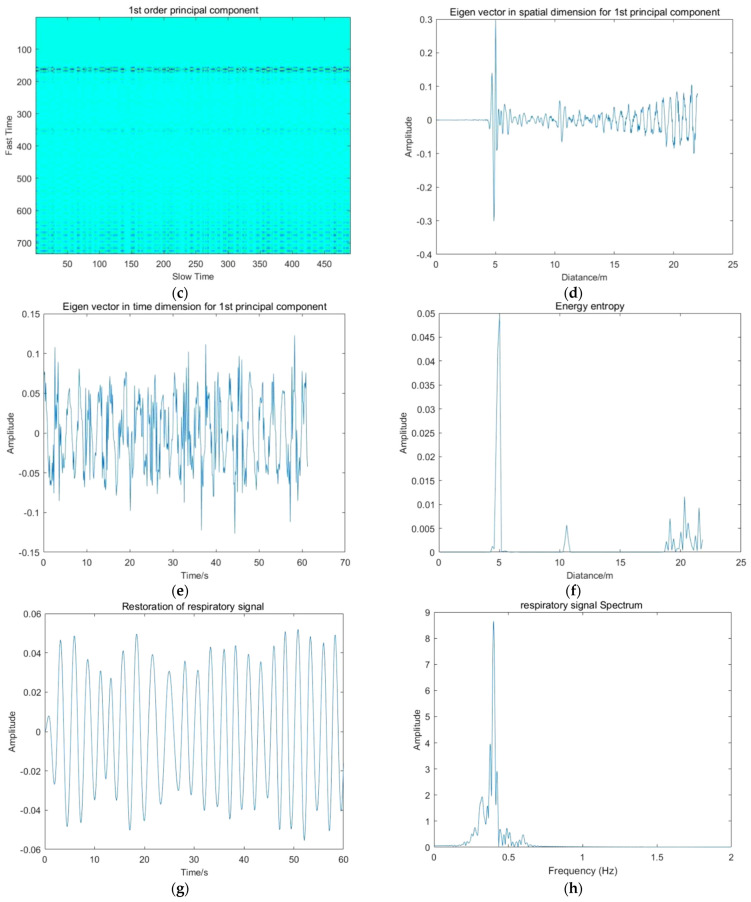

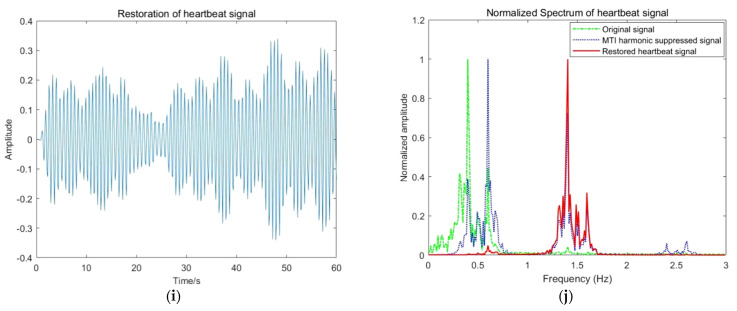
Results of essential steps for experiment group 5: (**a**) Radar raw data; (**b**) Radar data after preprocessing; (**c**) The relative principal component; (**d**) Spatial eigenvector; (**e**) Temporal eigenvector; (**f**) Energy entropy; (**g**) Restoration of respiratory signal; (**h**) Respiratory signal spectrum; (**i**) Restoration of heartbeat signal; (**j**) Heartbeat signal spectrum.

**Figure 29:**
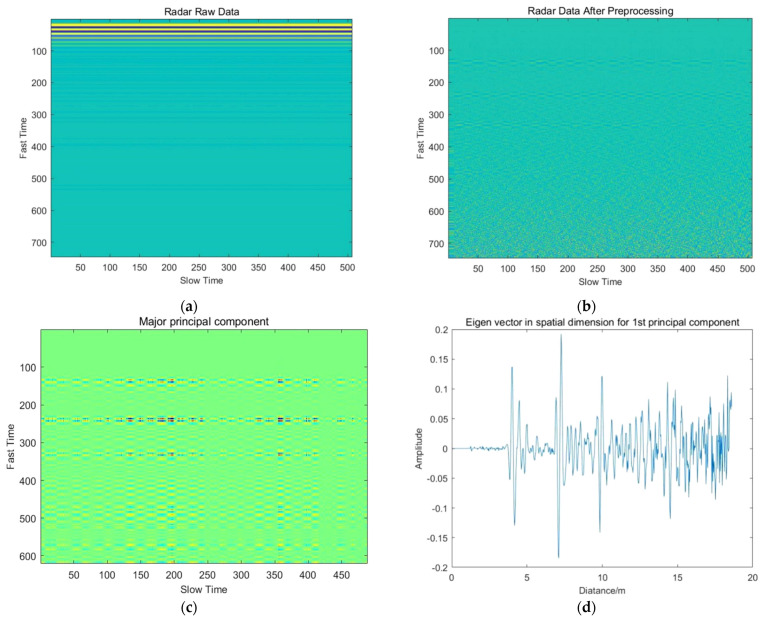

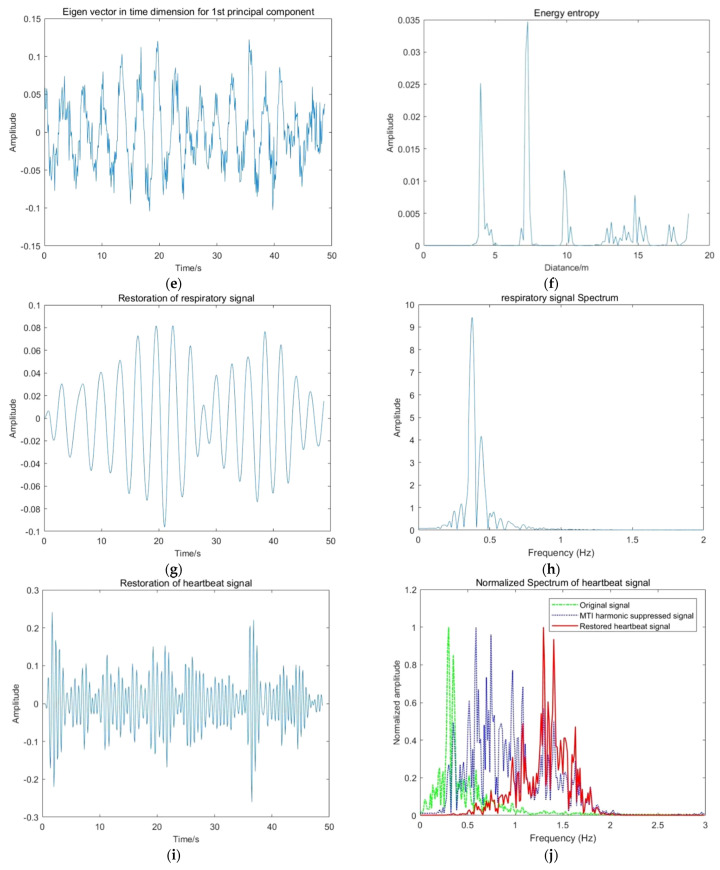
Results of essential steps for experiment group 6: (**a**) Radar raw data; (**b**) Radar data after preprocessing; (**c**) The relative principal component; (**d**) Spatial eigenvector; (**e**) Temporal eigenvector; (**f**) Energy entropy; (**g**) Restoration of respiratory signal; (**h**) Respiratory signal spectrum; (**i**) Restoration of heartbeat signal; (**j**) Heartbeat signal spectrum.

The results of essential steps for different experiment groups are shown in Figure 25, Figure 26, Figure 27, Figure 28 and Figure 29.

In order to illustrate the advancement of the proposed method in this paper, Table 7 compares some performances of the algorithm relative to the reference methods. (The performance of the algorithms is measured on the same experimental platform using the same experimental data, and the reference algorithms are reproduced by the author of this paper).

In order to further verify the effectiveness of the algorithm proposed in this paper in complex environments, the detected person (combinations of persons) and the corresponding positional relationship were randomly selected for the above 6 groups, and 15 verification experiments were performed for each group. According to the experimental results, one can summarize the key indicators as shown in Table 8.

Table 7 summarizes the results of multiple experiment groups when executing the proposed method and different algorithms, such as traditional FFT, VMD [27] and PE-EEMD [39]. In simple experimental scenarios, various methods can obtain accurate results, the proposed method can greatly shorten the calculation time and improve the timeliness by about 10 times, and at the same time, its detection accuracy and signal-to-noise ratio (SNR) are generally better than other algorithms. In complex experimental scenarios, such as long-distance, multiple targets, obstacle crossing, dynamic targets and so on, the performance of the proposed algorithm is particularly outstanding. For example, in group 4, the radar signal of a distant target covered by an obstacle is too weak; therefore, traditional algorithms have difficulty separating it from interference noise and clutter. However, the proposed method is still capable to obtain the information from the ninth higher order principal component based on the good characteristics of the SVD algorithm. Similarly, in the case of the multi-target detection of groups 5 and 6, the proposed method can successfully extract the information of a distant target interfered by the front targets. The detection of a heartbeat signal and dynamic target information is also more accurate and sensitive. In addition, most traditional algorithms need to traverse all data. In contrast, the proposed method adopts the principle of macroscopic big data analysis, after decomposing the temporal and spatial eigenvectors, it only processes the one-dimensional vectors, so it can always maintain high detection efficiency. Table 8 shows the statistical results of a large number of validation experiments, they reflect that the algorithm proposed in this paper can generally maintain effectiveness and high accuracy. For relatively simple scenarios, the algorithm in this paper can ensure a 100% success rate. For the long-range target detection through obstacles and the simultaneous detection of three different range targets, the vital signs are weak and subject to a lot of interference, but the algorithm in this paper can still ensure the success rate of 73.3% and 86.7%, with high detection accuracy and timeliness. In general, from the performance indicators such as accuracy, success rate, signal-to-noise ratio, and running time, one can conclude that the proposed algorithm has the properties of high efficiency, high precision, high sensitivity and high signal-to-noise ratio.

## 8. Conclusions

In this paper, a novel vital sign detection and extraction method based on Singular Value Decomposition (SVD) and Wavelet Transform decomposition is proposed. The experimental platform is established based on NVA6100 pulse radar system, the detection, acquisition and processing of UWB echo signals can be realized. The radar matrix contains discrete time series of *M* pulse signals after sampling; therefore, a two-dimensional matrix of *N* rows and *M* columns is formed. In order to improve the detection accuracy of vital signs in low signal-to-noise ratio environment, the algorithm proposed includes the following steps: signal preprocessing, signal denoising and decomposition; vital signs extraction and restoration; and vital signs reconstruction.

In the signal preprocessing, the clutter is suppressed with the Range Profile Subtraction method and the Time Mean Subtraction method, and the target signals are enhanced by the Advance Normalization method and Auto Gain Control.

Singular Value Decomposition (SVD) method is adapted to process the radar data after signal preprocessing, so as to achieve the purpose of denoising, data compression, dimension reduction and characteristic decomposition. The first six principal components are retained, and the temporal and spatial eigenvectors of each principal component of the radar signal are obtained.

The principal components extracted by SVD are screened based on energy proportion analysis. This paper adopted *sym6* wavelet base to perform wavelet transformation, the number of wavelet decomposition layers is set to seven. The hard threshold function is used to denoise the spatial eigenvectors *u_i_*, and the soft threshold function is used to denoise the temporal eigenvectors *v_i_*. Then, the energy entropy of each order principal component at different positions is calculated, and the maximum energy entropy is considered to be the location of the human body. The respiratory signal is restored through linear trend suppression and the Butterworth Filter, and the heartbeat signal is restored through the MTI harmonic canceller and the Butterworth Filter.

According to the above research works, the vital signs are reconstructed, and the results are analyzed. By setting up various complex experimental scenarios, such as long-distance, multiple targets, obstacle crossing, dynamic targets and so on, the performance of the proposed method is compared with the traditional FFT, VMD and PE-EEMD methods. From the performance indicators such as accuracy, success rate, signal-to-noise ratio (SNR) and running time, one can conclude that the proposed algorithm has the properties of high efficiency, high precision, high sensitivity and high signal-to-noise ratio.

## Figures and Tables

**Table 1:** Survivor location information based on energy entropy.

Order	Singular Value Size	Calculated Target Position (m)	*K*
1	170.04	0.810	97.1%
3	40.38	0.810	91.4%
5	19.17	0.821	80.2%

**Table 2:** Respiratory signal restoration result.

Order	Singular Value Size	Respiratory Frequency (Hz)	SNR (dB)
1	170.04	0.342	1.457
3	40.38	0.328	−4.269
5	19.17	0.340	−1.196

**Table 3:** Heartbeat signal restoration result.

Order	Singular Value Size	Respiratory Frequency (Hz)	SNR (dB)
3	40.38	1.375	−5.573

**Table 4:** Results and performance of the proposed algorithm.

Parameter	Result	Truth Value	Error	SNR (dB)
Position (m)	0.811	0.8	0.011	\
RF (Hz)	0.342	0.367	0.025	1.457
HF (Hz)	1.375	1.267	0.108	−5.573
Running time (s)	2.18	\	\	\

**Table 5:** Detection results for different subjects.

Subject	Position	RF		HF		Time (s)
	Error (m)	Error (Hz)	SNR (dB)	Error (Hz)	SNR (dB)	
A	0.011	0.025	1.457	0.108	−5.573	2.18
B	0.027	0.036	1.387	0.098	−3.342	1.95
C	0.018	0.017	1.255	0.148	−3.189	2.25
D	0.042	0.032	2.176	0.067	−3.476	2.17
E	0.023	0.038	1.167	0.147	−3.868	2.09

**Table 6:** Settings of different experimental groups.

No.	Objects	Distance (m)	State	Obstacle	Environment
1	A	0.8	Static	No	Indoor
2	B	4	Static	No	Outdoor
3	A	3	Static	Concrete (25 cm)	Indoor
4	C	11	Static	Concrete (32 cm)	Outdoor
5	A	5	Static	No	Outdoor
	B	10	Static		
6	A	4	Static	No	Outdoor
	B	7	Dynamic		
	C	10	Static		

**Table 7:** Results and performance comparison with the referenced algorithm.

No.	Value	Method																			
		FFT				VMD				PE-EEMD				Proposed				Measured			
1	Position(m)	0.89				0.87				0.84				0.811				0.8			
	Err(m)	0.09				0.07				0.04				0.011				\			
	RF(Hz)	0.36				0.36				0.34				0.342				0.37			
	SNR(dB)	−1.89				0.38				1.13				1.457				\			
	HF(Hz)	\				\				1.40				1.375				1.27			
	SNR(dB)	\				\				−5.24				−5.573				\			
	Time(s)	21.86				45.34				20.97				2.18				\			
2	Position(m)	4.26				4.12				4.14				4.075				4			
	Err(m)	0.26				0.12				0.14				0.075				\			
	RF(Hz)	0.37				0.34				0.34				0.336				0.32			
	SNR(dB)	−2.73				−1.08				−0.39				−0.083				\			
	HF(Hz)	1.19				1.32				1.34				1.404				1.38			
	SNR(dB)	−4.63				−4.11				−3.88				−3.268				\			
	Time(s)	27.79				50.38				26.89				2.76				\			
3	Position(m)	2.71				2.74				2.83				2.972				3			
	Err(m)	0.29				0.26				0.17				0.028				\			
	RF(Hz)	0.33				0.34				0.37				0.364				0.35			
	SNR(dB)	−2.48				−1.39				−0.87				0.553				\			
	HF(Hz)	\				1.34				1.34				1.376				1.39			
	SNR(dB)	\				−5.76				−5.07				−4.373				\			
	Time(s)	23.65				45.14				25.57				2.39				\			
4	Position(m)	\				\				\				11.774				11			
	Err(m)	\				\				\				0.774				\			
	RF(Hz)	\				\				\				0.369				0.37			
	SNR(dB)	\				\				\				−2.953				\			
	HF(Hz)	\				\				\				1.471				1.38			
	SNR(dB)	\				\				\				−4.475				\			
	Time (s)	23.67				49.76				28.78				2.93				\			
5	Position(m)	4.98		\		5.06		\		5.07		\		5.025		10.583		5		10	
	Err(m)	0.02		\		0.06		\		0.07		\		0.025		0.583		\			
	RF(Hz)	0.34		\		0.37		\		0.36		\		0.386		0.386		0.38		0.37	
	SNR(dB)	−4.22		\		−1.67		\		−1.79		\		−1.764		−1.764		\			
	HF(Hz)	\		\		1.43		\		1.40		\		1.404		1.404		1.42		1.38	
	SNR(dB)	\		\		−3.76		\		−3.36		\		−2.873		−2.873		\			
	Time(s)	28.76				56.87				31.84				3.08				\			
6	Position(m)	3.92	7.28		\	4.01	7.34		\	3.87	7.10		\	3.975	7.264		9.669	4	7		10
	Err(m)	0.08	0.28		\	0.01	0.34		\	0.13	0.10		\	0.025	0.264		0.331	\			
	RF(Hz)	0.32	0.45		\	0.35	0.42		\	0.32	0.39		\	0.366	0.441		0.366	0.37	0.47		0.35
	SNR(dB)	−3.87	−3.64		\	−1.41	−2.73		\	−2.03	−1.97		\	−0.887	−1.576		−0.887	\			
	HF(Hz)	\	\		\	1.23	\		\	1.21	1.17		\	1.296	1.632		1.404	1.35	1.89		1.38
	SNR(dB)	\	\		\	−5.87	\		\	−5.37	−5.43		\	−4.771	−5.315		−4.891	\			
	Time(s)	28.64				57.37				30.65				3.27				\			

**Table 8:** Key indicators of verification experiments.

No.	Position Err. (m)		RF Err. (Hz)		HF Err. (Hz)		Time (s)		Success Rate
	Max.	Avg.	Max.	Avg.	Max.	Avg.	Max.	Avg.	
1	0.042	0.025	0.038	0.027	0.148	0.105	2.25	2.16	100%
2	0.075	0.047	0.022	0.017	0.117	0.096	2.41	2.24	100%
3	0.037	0.031	0.026	0.018	0.084	0.047	2.39	2.07	100%
4	1.261	0.984	0.043	0.027	0.145	0.112	2.94	2.86	73.3%
5	0.632	0.216	0.038	0.021	0.145	0.097	3.08	2.79	100%
6	0.527	0.197	0.029	0.011	0.369	0.184	3.35	3.21	86.7%

## Data Availability

Not applicable.
